# Predicting the incidence of COVID-19 using data mining

**DOI:** 10.1186/s12889-021-11058-3

**Published:** 2021-06-07

**Authors:** Fatemeh Ahouz, Amin Golabpour

**Affiliations:** 1 Department of Computer Engineering, School of Engineering, Behbahan Khatam Alanbia University of Technology, Behbahan, Iran; 2 School of Medicine, Shahroud University of Medical Sciences, Shahroud, Iran

**Keywords:** COVID-19, Predicting, Data mining, Prevalence

## Abstract

**Background:**

The high prevalence of COVID-19 has made it a new pandemic. Predicting both its prevalence and incidence throughout the world is crucial to help health professionals make key decisions. In this study, we aim to predict the incidence of COVID-19 within a two-week period to better manage the disease.

**Methods:**

The COVID-19 datasets provided by Johns Hopkins University, contain information on COVID-19 cases in different geographic regions since January 22, 2020 and are updated daily. Data from 252 such regions were analyzed as of March 29, 2020, with 17,136 records and 4 variables, namely latitude, longitude, date, and records. In order to design the incidence pattern for each geographic region, the information was utilized on the region and its neighboring areas gathered 2 weeks prior to the designing. Then, a model was developed to predict the incidence rate for the coming 2 weeks via a Least-Square Boosting Classification algorithm.

**Results:**

The model was presented for three groups based on the incidence rate: less than 200, between 200 and 1000, and above 1000. The mean absolute error of model evaluation were 4.71, 8.54, and 6.13%, respectively. Also, comparing the forecast results with the actual values in the period in question showed that the proposed model predicted the number of globally confirmed cases of COVID-19 with a very high accuracy of 98.45%.

**Conclusion:**

Using data from different geographical regions within a country and discovering the pattern of prevalence in a region and its neighboring areas, our boosting-based model was able to accurately predict the incidence of COVID-19 within a two-week period.

**Supplementary Information:**

The online version contains supplementary material available at 10.1186/s12889-021-11058-3.

## Background

On December 8, 2019 the Chinese government reported the death of one patient and hospitalization of 41 others with unknown etiology in Wuhan [1]. This cluster initiated the novel coronavirus (COVID-19) epidemic respiratory disease. While the early cases were linked to the wet market, human-to-human transmission had led to widespread outbreak of the virus nationwide [2]. On January 30, 2020 the World Health Organization (WHO) declared COVID-19 as a public health emergency with international concern (PHEIC) [3].

On the basis of the global spread and severity of the disease, on March 11, 2020 the Director-General of WHO officially declared the COVID-19 outbreak a *pandemic* [4]. The pandemic as such, entered a new stage with rapid spread in countries outside China [5]. According to the 56th WHO situation report [6], as of March 16, 2020 the number of COVID-19 confirmed cases outside China exceeded those inside. Consequently, after March 17, 2020 WHO began to report the number of confirmed and dead cases on each continent as opposed to merely providing patient statistics in and out of China.

According to the 70th WHO situation report [7], by March 30, 2020 the number of people infected with COVID-19 worldwide were 693,282. 392,815 (about 57%) of whom were in Europe, 142,081 (about 20%) in the Americas, 103,775 (about 15%) in Western Pacific, 46,329 (about 7%) in Eastern Mediterranean, 4084 (about 0.5%) in South-East Asia, and 3486 (about 0.5%) in Africa. Of that total, 33,106 died worldwide, 23,962 of whom (around 72% of all death) were in Europe, 3649 (around 11%) in Western Pacific, and 5488 (around 17%) were in other regions collectively.

Due to the growing prevalence of COVID-19 across the world, several works have examined different aspects of the disease. They involve identifying the source of the virus as well as analyzing its gene sequences [8, 9], patient information [10], early cases in the countries infected [11–13], methods of virus detection [14, 15], the epidemiological outbreak [16, 17], and predicting COVID-19 cases [2, 17–20].

In [18], using heuristic method and WHO situation reports, an exponential curve was proposed to predict the number of cases in the next 2 weeks by March 30, 2020. The model was then tested for the 58th situation report. The authors reported 1.29% error. Afterwards, on the assumption that the current trend could continue for the next 17 days, they predicted that by March 30, 1 million cases outside China would be reported in the 70/71th WHO situation report. Given that the number of confirmed cases outside China was 693,176 on March 30 [21], their forecast error was 44.26%.

In [17], the CoronaTracker team proposed a Susceptible-Exposed-Infectious-Recovered (SEIR) model based on the queried data in their website, and made the 240-day prediction of COVID-19 cases in and out of China, started on 20 January 2020. They predicted that the outbreak would reach its peak on May 23, 2020 and the maximum number of infected individuals would amount to 425.066 million globally. In addition, the authors stated that this number would start to drop around early July 2020 and reach below 10,000 on 14 Sep 2020. Given the information available now, these predictions were far from what really happened around the world.

Elsewhere [19], the authors examined some available models to predict 5 and 10-day ahead of cumulative cases in Guangdong and Zhejiang by February 23, 2020. They used generalized logistic growth, the Richards growth, and a sub-epidemic wave model, which were utilized to forecast some previous infectious outbreaks.

Although some works have proposed methods for predicting COVID-19 cases, to our knowledge at the time of writing this paper, none have been comprehensive and have not predicted the new cases in each geographical region along with each continent. In this study, using the COVID-19 Cases dataset provided by Johns Hopkins University [22], we aim to predict COVID-19 infected people in each geographical regions included in the dataset as well as each continent in the coming 2-week period. Predicting the situation in the current pandemic is very crucial to containment of the threat because it helps make timely medical measures e.g. equipping medical facilities, managing resource allocation, sending more personnel to high-risk areas, deciding whether to close borders or resume traffic, and suspending or resuming community services.

## Methods

### Dataset

COVID-19 epidemiological data have been compiled by the Johns Hopkins University Center for Systems Science and Engineering (JHU CCSE) [22]. The data have been provided in three separate datasets for confirmed, recovered, and death cases since January 22, 2020 and are updated daily. In each of these datasets, there is a record (row) for every geographic region. The variables in each dataset are province/state, country/region, latitude, longitude, and the incremental dates since January 22. For each region, the value for any date indicates the cumulative number of confirmed/recovered/death cases from January 22, 2020.

In this study, according to the input requirements of the proposed model, we changed the data representation so that instead of three separate datasets for three groups of confirmed, recovered, and death cases, only one dataset containing the information of all three groups was arranged. In this new dataset, each record (or row) of the dataset contains information about the number of confirmed, recovered, or deaths per day for each geographic region. As a result, the variables in this new dataset are: Province / State, Country / Region, Latitude (Lat), Longitude (Long), Date (specifying a certain date), Cases (indicating the number of confirmed, recovered, or death cases on the certain date), and Type (specifying the type of cases, i.e. confirmed, recovered, or death) as suggested by Rami Krispin [23].

In this study, the data were applied into the analysis by March 29, 2020, with 50,660 records and 7 variables. This period includes information about parts of winter and spring in the northern hemisphere and summer and autumn in the southern hemisphere. By March 29, the dataset consisted of cases from 177 countries and 252 different regions around the world. There were 720,139 confirmed, 33,925 death, and 149,082 recovered cases in the dataset.

### Preprocessing step

**Fig. 1:**
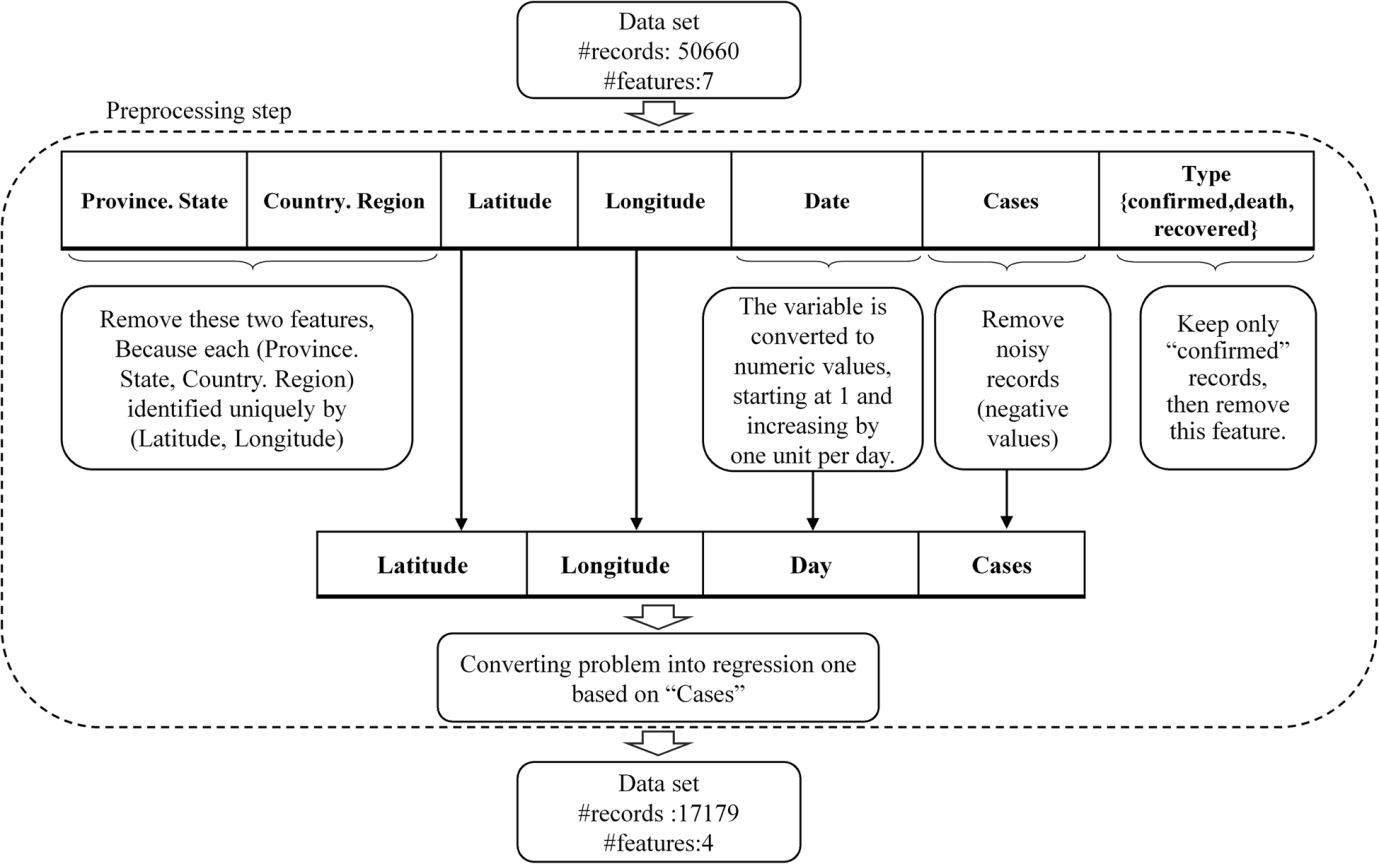
Preprocessing steps on COVID-19 dataset

Pre-processing was carried out on the dataset before training the proposed model. Figure 1 shows the preprocessing steps. The dataset was first examined for noise, since the noise data were considered as having negative values in Cases variable. The dataset contained 42 negative values in this variable. After deleting these values, the number of records were reduced to 50,618.

Subsequently, the Date variable was written in numerical format and renamed into “Day” variable. To that effect, January 22, 2020 marked the beginning of the outbreak and the next days were calculated in terms of distance from the origin. As a result, January 22 and March 29 were considered as Day 1 and Day 68, respectively.

Since each region is uniquely identified by its latitude and longitude, the data for Province/State and Country/Region were excluded from the dataset. Moreover, as the study aimed at predicting the incidence in any geographical region, we considered only those records providing information on the confirmed cases (17,179 records), but not on the dead or the recovered. So, after preserving the records with “Confirmed” value in the Type variable, it was deleted from the dataset. In this study, the “Cases” is considered as the dependent variable.

### Constructing the prediction model

An ensemble method of regression learners was utilized to predict the incidence of COVID-19 in different regions. The idea of ensemble learning is to build a prediction model by combining the strengths of a collection of simpler base models called weak learners [24]. At every step, the ensemble fits a new learner to the difference between the observed response and the aggregated prediction of all learners grown previously. One of the most commonly used loss functions is least-squares (LS) error [25].

In this study, the model employed a set of individual Least-squares boosting (LSBoost) learners trying to minimize the mean squared error (MSE). The output of the model in step m, F_m_(x), was calculated using Eq. 1:

1 $$ {F}_m(x)={F}_{m-1}(x)+{\rho}_mh\left(x;{a}_m\right) $$

where x is input variable and h(x;a) is the parameterized function of x, characterized by parameters a [25]. The values of ρ and a were obtained from Eq. 2:

2 $$ \left({\rho}_m,{a}_m\right)=\arg {\mathit{\min}}_{a,\rho}\sum \limits_{i=1}^N{\left[{\tilde{y}}_i-\rho h\left({x}_i;a\right)\right]}^2 $$

Where N is the number of training data and $\tilde{y}_{i}$ is the difference between the observed response and the aggregated prediction up to the previous step.

**Fig. 2:**
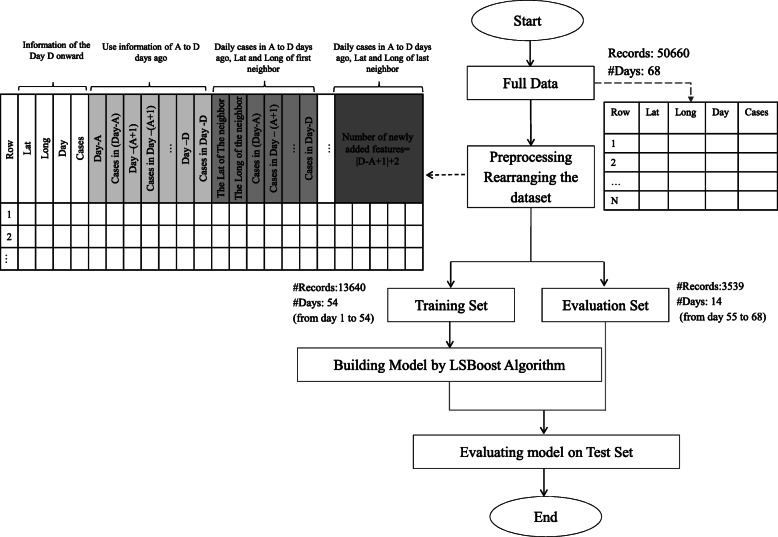
The structure of the Proposed model

Due to the recent major changes in the incidence of COVID-19 worldwide over the past 2 weeks, we aimed to predict the number of new cases as an indicator of prevalence over the next 2 weeks. The structure of the proposed method is shown in Fig. 2.

Since the incubation period of COVID-19 can be 14 days [26], we assumed that we needed at least 14 days prior information to predict the incidence of Covid-19 in 1 day. Therefore, the proposed model examined all possible intervals between the first and the last 14 days to find the optimal time period to use its information to predict the number of cases in the coming days.

We hypothesized that the incidence in any region might follow the pattern of recent days in the same region and nearby. Therefore, after determining the optimal time period, the model added the information on confirmed cases in each region and nearby in the specified period to the same region’s record in the dataset.

After setting the time interval, [A, B], and the number of neighbors, the dataset was rearranged. In this case, the number of records was reduced from N to M, where M is calculated from Eq. 3:

3 $$ M=N-\left(B\times R\right) $$

Where R is the number of different regions in the dataset and B is the last day of the time period. Similarly, the number of variables stored for each record increased from the first 4 variables (latitude, longitude, Day and Cases) to F, which is calculated from Eq. 4:

4 $$ F=4+2\times \left|B-A+1\right|+ NN\times \left(|B-A+1|+2\right) $$

Where NN is the number of neighbors and 4 is the number of variables in the original data set because for each geographical region, Lat, Long, Day and Cases are stored. |B-A + 1| is the number of days within the period that participate in the forecast of the next 14 days. The value of NN is multiplied by 2 because for each neighbor, latitude and longitude are added to the record information. Furthermore, for each day within the period of forecast, the Cases were added to the record information, so NN was multiplied by|B-A + 1|. For each region, the Day and Cases data during the period were added as well. Thus, |B-A + 1| was multiplied by 2. It should be noted, however, that the dependent variable remained the Cases of current day.

Since the number of both the nearby regions and the previous days effective in forecasting were unknown, we assumed these values to be unknown variables and obtained the most accurate model by examining all possible combinations of such variables in an iterative process.

The accuracy of the model was evaluated in terms of Mean Squared Error (MSE) and Mean Absolute Error (MAE); Due to the normalization of MAE between [0, 1], the evaluation error is equal to 2 times MAE. To do so, the information of the last 2 weeks on all regions was considered as a validation set, and the model was trained using other information in the dataset.

### Forecast incidence in the next 2 weeks

A new test set was created to predict incidence in the next 2 weeks (by April 12, 2020). The number of records in this dataset was equal to that of unique geographical regions in the COVID-19 dataset. Then, according to the best neighborhood and optimal time interval specified in the previous step, the necessary features were provided for each record. After that, the best model was created in the previous step was retrained on the entire dataset as a training set. Later on, this model was examined on the new test set to predict the incidence rate.

### Evaluation the actual performance of the proposed model

Given that the actual number of confirmed cases within March 30–April 12, 2020 period was available at the time of review, the performance of the proposed model was measured based on percent error between the predicted and the actual values. The percent error was calculated from Eq. 5:

5 $$ \delta =\left(\frac{\left|{v}_A-{v}_E\right|}{v_A}\right)\times 100 $$

Where δ is percent error, *v*_*A*_ is the actual observed value and *v*_*E*_ is the expected (predicted) value. Furthermore, according to the predicted and actual confirmed cases in 252 geographical regions in the dataset, the continental incidence rate was calculated using Eq. 6:

6 $$ Continental\ incidence\ rate=\left(\frac{I_C}{I_W}\right)\times 100 $$

where I_C_ is the incidence in each continent and I_W_ is the global incidence of COVID-19 from March 30 to April 12, 2020.

The experimentation platform is Intel® Core™ i7-8550U CPU @ 1.80GHz 1.99 GHz CPU and 12.0 GB of RAM running 64-bits OS of MS Windows. The pre-processing and model construction has been implemented in MATLAB.

## Results

### Model construction

The number of neighbors ranged from zero to 10. The value of 10 was obtained by trial and error. Euclidean distance based on latitude and longitude was used to calculate nearest neighbors. Given that the dataset contains data from January 22, 2020 to March 29, 2020 for the day we want to predict the incidence, the nearest and farthest days were selected as 14 and 54, respectively. Because the number of confirmed cases varies greatly from region to region, the proposed algorithm was implemented for 3 different groups of regions: for regions with less than 200 confirmed cases per day (16,825 records), those with 200 to 1000 cases per day (220 records), and those with over 1000 cases per day (152 records).

Table 1 shows the results of the best proposed model with regard to the different composition of the neighborhood and the days before. In order to predict the incidence of COVID-19 in regions with more than 1000 confirmed cases per day, the proposed model demonstrated the best performance with MAE of 6.13%, considering the information of the last 14 to 17 days of the region and its two neighboring areas. In the dataset, the number of cases records in these regions varied from 1019 to 19,821.

**Table 1:** The results of the best models evaluated on COVID-19 dataset (January 22, 2020 to March 29, 2020)

Maximum number of confirmed cases in a day		Number of Neighbors	Interval of days [min, max]	MSE		MAE	
				Value	Percent	Value	Percent
< 200	Train	–	[14,34]	1.86	0.005%	0.52	0.29%
	Test			407.47	1.04%	9.12	4.71%
[200,1000)	Train	9	[14, 20]	1.71	0.002%	0.62	0.07%
	Test			1.59e+ 04	1.87%	79.01	8.54%
≥1000	Train	2	[14, 17]	140.62	0.00003%	5.89	0.03%
	Test			7.14e+ 06	1.79%	1.2e+ 03	6.13%

For regions with 200 to 1000 cases per day, the proposed model performed best with respect to the 9 nearest neighboring areas and with data from the last 14 to 20 days, with MAE of 8.54% on the validation set. For regions with fewer than 200 cases per day, on the other hand, the proposed model performs best with MAE of 4.71%, taking into account the region data for the last 14 to 34 days.

### Prediction of incidence by April 12, 2020

**Fig. 3:**
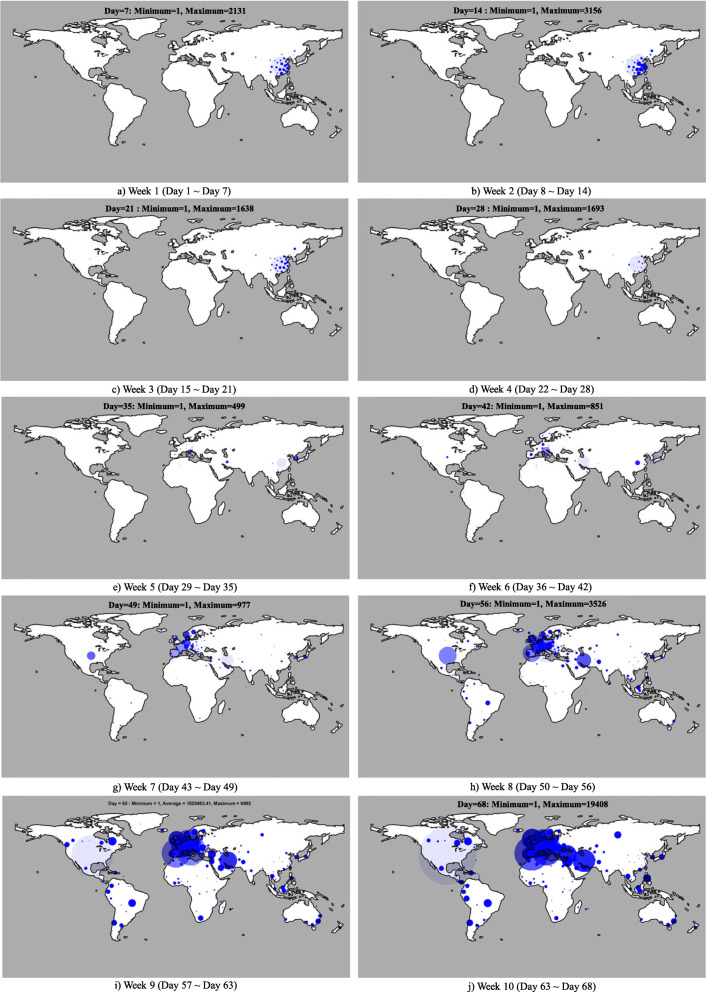
Visualize the outbreak over the days (created by ourselves, gimp software, open source)

Figure 3 shows the prevalence of the COVID-19 from the first week to the tenth week in different regions, based on the information provided by the COVID-19 epidemiological dataset [22]. In this Figure, the diameter of the circles is proportional to the prevalence in those regions and the center of each circle matches the geographical coordinates of the region.

Table 2 shows the results of the forecast as to the number of new cases per day on different continents. According to the location of the continents in the northern and southern hemispheres, the period in question contains winter and early spring information in the continents of North America, Europe and almost entire parts of Asia. It includes summer and parts of autumn in Australian and approximately whole South America. Given that Africa lies in all four hemispheres, the data recorded for this continent in this period in the data set includes all seasons.

**Table 2:** Forecast the COVID-19 new cases for the next 2 weeks

*Date*	*Continents*						*Total number of confirmed cases*
	Africa	Asia	Australian	Europe	North America	South America	
22 Jan ~ 29 Mar	4995	161,986	4522	385,097	150,877	11,740	719,217
30-Mar	635	7720	802	37,853	19,269	1906	68,185
31-Mar	820	7227	722	37,433	16,890	2000	65,092
1-Apr	472	7533	338	38,512	19,625	1508	67,988
2-Apr	1046	6438	981	44,047	18,435	1955	72,902
3-Apr	1047	6790	780	53,087	19,802	2359	83,865
4-Apr	1015	9739	872	51,954	19,302	2258	85,140
5-Apr	1014	10,563	1226	47,352	19,579	2490	82,224
6-Apr	1447	6867	1015	48,562	19,060	2530	79,481
7-Apr	1636	8027	1057	51,192	20,191	2768	84,871
8-Apr	2087	6786	1444	56,826	19,546	2550	89,239
9-Apr	2157	7749	1270	55,316	20,475	2685	89,652
10-Apr	1976	5818	1430	54,377	20,819	2573	86,993
11-Apr	1849	8962	1390	56,284	19,627	2351	90,463
12-Apr	1930	6781	1199	54,870	20,337	2806	87,923
Total	19,131	107,000	14,526	687,665	272,957	32,739	1,134,018
Prevalence growth rate	**283.00**	−33.94	221.23	78.57	80.91	178.87	57.67

By April 12, 1,134,018 new cases worldwide were expected to be on record. Of these, Europe with 687,665 (60.64%), North America with 272,957 (24.07%) and Asia with 107,000 (9.44%) new cases were the most prevalent, whereas Australia with 14,526 (1.28%), Africa with 19,131 (1.69%) and South America with 32.739 (2.89%) new cases were the least incidence. Africa, Europe and South America had the highest rates of COVID-19 incidence, with 283, 221.23, and 178.87%, respectively. Asia was the only continent that had slowed its growth with an incidence rate of − 34.

**Fig. 4:**
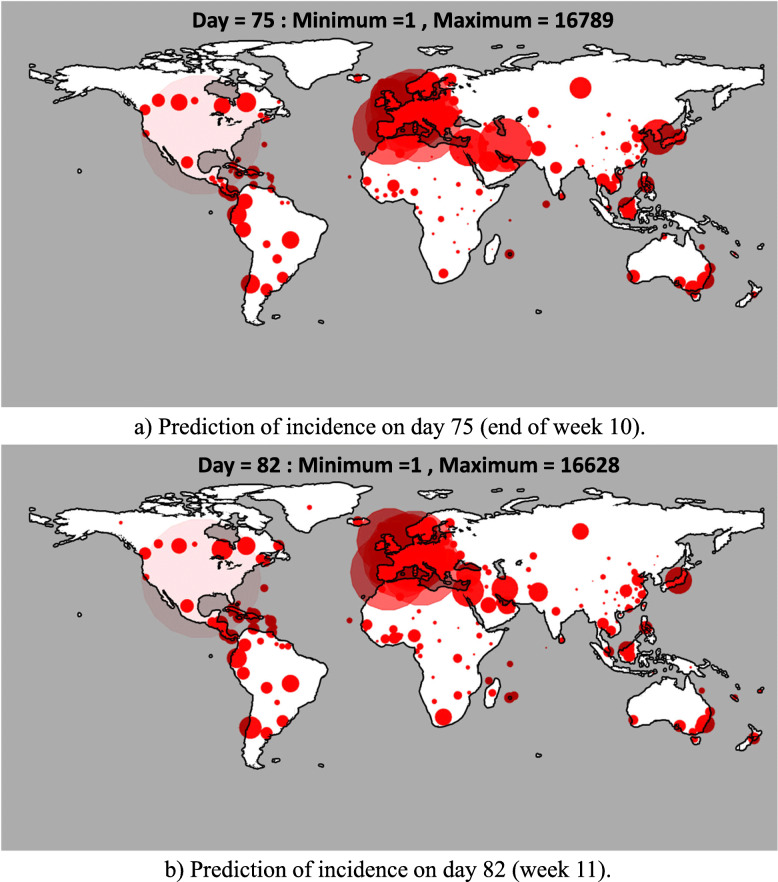
Prediction of the incidence in week 10 and 11 (created by ourselves, gimp software, open source)

Figure 4 shows the prediction of incidence rates in different regions. Accordingly, the prevalence would decrease over the next 2 weeks in the Middle East, yet it would increase in North America and Europe. Outbreak forecasts for 244 geographic regions are provided in Additional file 1: Appendix 1.

### Comparison of predicted and actual cases from March 30 to April 12, 2020

**Fig. 5:**
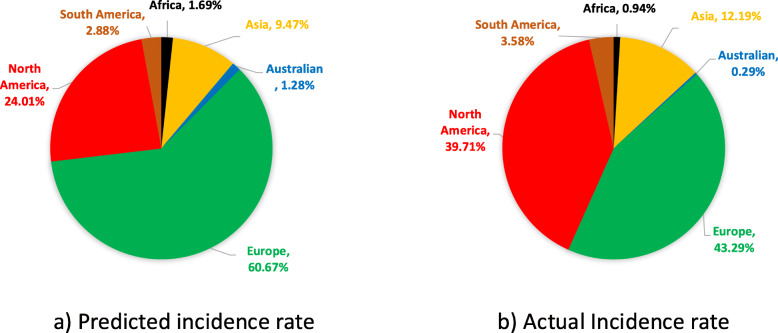
Comparison of predicted and actual continental incidence rates between March 30 and April 12, 2020

Table 3 shows the total number of daily cases in the 252 regions surveyed between March 30 and April 12, 2020. As shown, the daily percent error is below 20%. The best accuracy of the proposed model in predicting the incidence of COVID-19 was obtained on April 10 with 99.6%, and the worst on April 11 with 81.3%. Data analysis of the two-week continental incidence rates are also shown in Fig. 5. The best predicted continental incidence rates were found in South America and Asia with 18.15 and 21.04% percent error, respectively. The worst cases, still, were observed in Africa and Australian with more than 80% percent errors.

**Table 3:** Comparison of predicted and actual daily incidence of COVID-19

Date	Across all 252 geographic regions		Percent error
	Predicted	Actual	
30-Mar	68,185	65,321	4.38%
31-Mar	65,092	76,799	15.24%
1-Apr	67,988	76,657	11.31%
2-Apr	72,902	81,340	10.37%
3-Apr	83,865	83,272	0.71%
4-Apr	85,140	80,392	5.91%
5-Apr	82,224	71,994	14.21%
6-Apr	79,481	73,285	8.45%
7-Apr	84,871	77,773	9.13%
8-Apr	89,239	84,275	5.89%
9-Apr	89,652	86,461	3.69%
10-Apr	86,993	87,520	0.60%
11-Apr	90,463	76,217	18.69%
12-Apr	87,923	95,353	7.79%
Total number of confirmed cases	1,134,018	1,116,659	1.55%

## Discussion

Data mining is capable of presenting a predictive model and extracting new knowledge from retrospective data. The way data is processed, as well as the variables selected, had a significant impact on knowledge discovery. There are various data mining techniques used to predict an outbreak. As an actual global health concern, COVID-19 had already developed into one of the world’s major emergencies. The present study proposed to investigate its outbreak worldwide during a two-week period via a predictive model based on retrospective data. It was concluded that such a model could be presented with acceptable error rates.

The study made use of a coronavirus dataset to design an incidence of COVID-19 prediction model. According to the incidence rate per day, the model was trained based on three groups of below 200, 200–1000 and above 1000 cases. One-way ANOVA results showed that there was a statistically significant difference between the prevalence rates in the three groups (*p*-value < 0.001). For each group, the prediction model was implemented and the incidence was predicted for the next 2 weeks. The proposed model achieved about 10% error (90% accuracy) for the group of less than 200 cases, 18% error (82% accuracy) for the group of 200–1000 cases, and 13% error (87% accuracy) for that exceeding 1000 cases.

In this study, as the incidence of COVID-19 was evaluated for 68 days worldwide, and a prediction model presented for the two-week period (i.e., March 30–April 12, 2020), more than 1000,000 people were expected to contract the disease within the next 2 weeks, which was statistically up 58% compared to 700,000 of the outbreak by March 29, 2020.

The study found that adjacent regions with a prevalence of less than 1000 had similar incidence, so the incidence of each of these regions could be determined from information on neighboring areas. The use of neighborhood information enables the model to indirectly consider the effective policies of other regions in predicting the incidence of COVID-19 in each region.

Given that the proposed model was trained using only 68-day data (which was the most up-to-date information at the time of writing), the accuracy of predicting the incidence above 81% was deemed acceptable for such an unknown disease. Further, according to the results shown in Table 3, the model prediction error for a total of 12 days for 252 regions was less than 2%. Therefore, if the data of each country were stored more precisely using more geographical regions, it was promising that we could create an accurate model for predicting the incidence of covid-19 over a two-week period in each country. While many unknowns would be expected of a new pandemic, having this information can guide planning and resource allocation for prevention, treatment, and palliative care.

Although time series usually need to be long enough (normally a few years) to adequately account for seasonality, based on the results of the model implementations, we believe that this model, even with that short a time series, is able to manage seasonality and can predict the number of cases with acceptable accuracy (see Additional file 1: Appendices 2 and 3 for the results of all analyses). However, it is suggested that future research specifically address the effect of seasonal changes on the prevalence of this disease.

One of the limitations of the study was that the dataset did not provide sufficient information from all continents. Given that the disease did not occur simultaneously on all continents, and the continental prevalence was in most cases after the 40th day of the first case in China, 68 days of data did not seem sufficient to predict the prevalence of such an unknown disease.

In Africa, the first case was reported in more than 80% of the 45 geographical regions since the 50th day. The number of confirmed cases since then was 4682, which was 97.83% of the total 4783 confirmed cases in Africa. In Australian, the first case was reported in more than 45% of the 11 geographical regions from the 40th day onwards. Also, out of a total of 4504 cases on the continent, 4478 cases (99.4%) were confirmed then.

In Europe, the first case was reported in 60 of the 69 geographic regions in the dataset from the 40th day onwards. Out of a total of 385,735 cases, information on 384,268 cases (i.e. 99.62%) has also been entered since that day. Similarly, South America confirmed its first case after the 40th day in 16 out of 17 regions. It is noteworthy that out of a total of 11,642 cases, 11,542 (14.99%) were confirmed from day 50 onwards.

In contrast, 88% of the North American regions had their first cases confirmed since day 50. In addition, of the 46 confirmed cases by March 29, 2020 on the continent, 38 were reported since day 50 (82.61%) And 41 were confirmed from day 40 onwards (89.13%).

Due to insufficient information on some continents as a result of their prevalence later than the declared beginning of the outbreak, the effect of measures such as increasing the number of tests taken per day as well as quarantine restrictions in some continents such as Europe, begin in place from March 30 to April 12, were not reflected in the dataset.

Nevertheless, the inaccurate prediction of the number of cases in Africa could be attributed, in turn, to the insufficient information about the continent in the dataset. In 80% of the African regions, the first confirmed case was recorded 50 days into the outbreak. Out of a total of 4786 cases there, up until the 68th day, 4682 cases (more than 97%) were reported since day 50.

In addition, due to the fact that latitude and longitude are two important indicators in the data set, the non-uniformity of recording these information for different geographical regions is another limitation of the work; for some areas, the information is about one state of a country and for some areas it is for the whole country. For example, in the dataset for USA, all cases are provided in terms of only one latitude and longitude, but for Netherlands, the data of COVID-19 cases are provided for four different latitude and longitude pairs.

Another limitation of this study was the use of data from all countries coping with in COVID-19 with their own protocols for testing and identifying patients. However, in general, this is the only global dataset for COVID-19 that has been used in other studies [16, 17]. Besides, early information on each country was taken into account in the proposed model to predict the incidence in that country to reduce the mentioned limitation.

It is worth noting that the model rests on both the info provided by the dataset and the current measures taken in dealing with the disease. Hence, if government’s’ policies to tackle the disease change, so will the accuracy of the information.

## Conclusions

Since epidemiological models such as SIR failed to accurately predict COVID-19 cases, as stated in [17, 27, 28], the current study relied on data from January 22 to March 29 provided by Johns Hopkins University and proposed a more complex model based on machine learning methods. The mean absolute error of the proposed model was 6.13% in predicting the incidence of COVID-19 in the two-week period of March 16–29 for regions with more than 1000 cases per day. The MAE was 8.45 and 4.71% for regions with a daily incidence rate between 200 and 1000 cases and less than 200 cases, respectively. An accuracy of more than 82% on the evaluation set confirms our perception that the pattern of incidence of a region is influenced by the pattern of disease in recent days in the same region and neighboring areas.

Last but not least, despite numerous limitations of the dataset, lack of knowledge about such an unknown disease and changes in disease control policies in different countries during the period under scrutiny, the proposed model proved effective in predicting the global incidence of COVID-19 in the two-week period of March 30 and April 12 with 98.45% accuracy. In addition, the accuracy of the proposed model in predicting daily cases in a worst-case scenario was 81.31%.

This model is written in general and can be run for different intervals (see Additional file 1: Appendix 4). It is suggested that the model be implemented for future data as well.

## Supplementary Information

**Additional file 1: Appendix 1.** Point-to-point forecast for all areas in the dataset. **Appendix 2.** Investigation the effect of seasonal changes on model performance. **Appendix 3.** The performance of the proposed method on randomly selected regions. **Appendix 4.** The results of the proposed method on the updated data.

## Data Availability

The dataset analyzed during the current study is public and it is available in the [https://data.humdata.org/dataset/novel-coronavirus-2019-ncov-cases] and in [https://codeload.github.com/RamiKrispin/coronavirus-csv/zip/master].

## Abbreviations

WHO: World Health Organization
PHEIC: Public Health Emergency with International Concern
SEIR: Susceptible-Exposed-Infectious-Recovered
JHUCCSE: Johns Hopkins University Center for Systems Science and Engineering
Lat: Latitude
Long: Longitude
LSBoost: Least-squares boosting
MSE: Mean Squared Error
MAE: Mean Absolute Error

## References

[1] Nkengasong J. Author Correction: China’s response to a novel coronavirus stands in stark contrast to the 2002 SARS outbreak response. Nat Med. 2020;26(3):441. 10.1038/s41591-020-0816-5. 10.1038/s41591-020-0816-5 PMC7095842 32139890

[2] Roosa K, Lee Y, Luo R, Kirpich A, Rothenberg R, Hyman JM, Yan P, Chowell G (2020). Real-time forecasts of the COVID-19 epidemic in China from February 5th to February 24th, 2020. Infect Dis Model.

[3] Eurosurveillance Editorial T (2020). Note from the editors: World Health Organization declares novel coronavirus (2019-nCoV) sixth public health emergency of international concern. Eurosurveillance.

[4] World Health Organization, WHO Director-General's opening remarks at the media briefing on COVID-19 - 11 March 2020. 2020. Available from: https://www.who.int/dg/speeches/detail/who-director-general-s-opening-remarks-at-the-media-briefing-on-covid-19%2D%2D-11-march-2020. Accessed 27 May 2021.

[5] Bedford J (2020). COVID-19: towards controlling of a pandemic.

[6] Who (2020). World Health Organization, Coronavirus disease 2019 (COVID-19) situation report −60.

[7] World Health Organization, Coronavirus disease 2019 (COVID-19) Situation Report −70. 2020 [updated 19March 2020. Available from: https://www.who.int/docs/default-source/coronaviruse/situation-reports/20200330-sitrep-70-covid-19.pdf?sfvrsn=7e0fe3f8_4. Accessed 27 May 2021.

[8] Ji W, Wang W, Zhao X, Zai J, Li X (2020). Cross-species transmission of the newly identified coronavirus 2019-nCoV. J Med Virol.

[9] Paraskevis D, Kostaki EG, Magiorkinis G, Panayiotakopoulos G, Sourvinos G, Tsiodras S (2020). Full-genome evolutionary analysis of the novel corona virus (2019-nCoV) rejects the hypothesis of emergence as a result of a recent recombination event. Infect Genet Evol.

[10] Huang C, Wang Y, Li X (2020). Clinical features of patients infected with 2019 novel coronavirus in Wuhan, China (vol 395, pg 497, 2020). Lancet.

[11] Kim JY, Choe PG, Oh Y, Oh KJ, Kim J, Park SJ, et al. The first case of 2019 novel coronavirus pneumonia imported into Korea from Wuhan, China: implication for infection prevention and control measures. J Korean Med Sci. 2020;35(5):e61. 10.3346/jkms.2020.35.e61. 10.3346/jkms.2020.35.e61 PMC7008073 32030925

[12] Bernard Stoecklin S, Rolland P, Silue Y, Mailles A, Campese C, Simondon A, et al. First cases of coronavirus disease 2019 (COVID-19) in France: surveillance, investigations and control measures, January 2020. Euro Surveill. 2020;25(6):2000094. 10.2807/1560-7917.ES.2020.25.6.2000094. 10.2807/1560-7917.ES.2020.25.6.2000094 PMC7029452 32070465

[13] Giovanetti M, Benvenuto D, Angeletti S, Ciccozzi M. The first two cases of 2019-nCoV in Italy: Where they come from? J Med Virol. 92(5):518–21. 10.1002/jmv.25699. 10.1002/jmv.25699 PMC7166327 32022275

[14] Corman VM (2020). Detection of 2019 novel coronavirus (2019-nCoV) by real-time RT-PCR. Eurosurveillance.

[15] Zhang NR (2020). Recent advances in the detection of respiratory virus infection in humans. J Med Virol.

[16] Dey SK, Rahman MM, Siddiqi UR, Howlader A. Analyzing the epidemiological outbreak of COVID-19: a visual exploratory data analysis approach. J Med Virol. 92(6):632–8. 10.1002/jmv.25743. 10.1002/jmv.25743 PMC7228278 32124990

[17] Binti Hamzah FA (2020). CoronaTracker: world-wide COVID-19 outbreak data analysis and prediction.

[18] Koczkodaj WW, Mansournia MA, Pedrycz W, Wolny-Dominiak A, Zabrodskii PF, Strzałka D, Armstrong T, Zolfaghari AH, Dębski M, Mazurek J (2020). 1,000,000 cases of COVID-19 outside of China: The date predicted by a simple heuristic. Glob Epidemiol.

[19] Roosa K, Lee Y, Luo R, Kirpich A, Rothenberg R, Hyman JM, et al. Short-term Forecasts of the COVID-19 Epidemic in Guangdong and Zhejiang, China: February 13–23, 2020. J Clin Med. 2020;9(2):596. 10.3390/jcm9020596. 10.3390/jcm9020596 PMC7073898 32098289

[20] Nishiura H, Jung SM, Linton NM, Kinoshita R, Yang YC, Hayashi K, et al. The extent of transmission of novel coronavirus in Wuhan, China, 2020. J Clin Med. 2020;9(2):330. 10.3390/jcm9020330. 10.3390/jcm9020330 PMC7073674 31991628

[21] Organization, W.H (2020). Coronavirus disease 2019 (COVID-19) Situation Report −70.

[22] (CCSE), J.H.U.C.f.S.S.a.E.J (2020). Novel Coronavirus (COVID-19) Cases Data.

[23] Krispin R (2020). Coronavirus.

[24] Hastie T, Tibshirani R, Friedman J (2008). The Elements of Statistical Learning, second edition.

[25] Friedman J. Greedy function approximation: a gradient boosting machine. Ann Stat. 2000;29:1189–232. 10.1214/aos/1013203451.

[26] Organization, w.H (2020). Transmission of SARS-CoV-2: implications for infection prevention precautions.

[27] Postnikov EB (2020). Estimation of COVID-19 dynamics “on a back-of-envelope”: Does the simplest SIR model provide quantitative parameters and predictions?. Chaos, Solitons Fractals.

[28] Cooper I, Mondal A, Antonopoulos CG (2020). A SIR model assumption for the spread of COVID-19 in different communities. Chaos, Solitons Fractals.

