# Supplementary material for: Predicting the incidence of COVID-19 using data mining
Source: BMC Public Health. 2021 Jun 7;21:1087. doi: 10.1186/s12889-021-11058-3 (PMC8182740; doi:10.1186/s12889-021-11058-3)
Supplement: Supplementary file 1 — Additional file 1: Appendix 1. Point-to-point forecast for all areas in the dataset. Appendix 2. Investigation the effect of seasonal changes on model performance. Appendix 3. The performance of the proposed method on randomly selected regions. Appendix 4. The results of the proposed method on the updated data. [file 12889_2021_11058_MOESM1_ESM.docx]

Appendices:

[Appendix 1: Point-to-point forecast for all areas in the dataset. 2](#_Toc71039046)

[Appendix 2: Investigation the effect of seasonal changes on model performance. 6](#_Toc71039047)

[Appendix 3: The performance of the proposed method on randomly selected regions. 9](#_Toc71039048)

[Appendix 4: The results of the proposed method on the updated data 13](#_Toc71039049)

# **Appendix 1:** Point-to-point forecast of the number of confirmed cases between March 30 and April 12 for all areas in the dataset at the time of submission.

| **Row** | **Continents** | **Longitude** | **Latitude** | **30 Mar** | |  |  |  |  | **6 Apr** | | |  |  |  | **12 Apr** | |
| --- | --- | --- | --- | --- | --- | --- | --- | --- | --- | --- | --- | --- | --- | --- | --- | --- | --- |
| 1 | Africa | -15.3101 | 13.4432 | 1 | 0 | 2 | 2 | 5 | 12 | 0 | 8 | 7 | 12 | 52 | 23 | 3 | 30 |
| 2 | Africa | -15.1804 | 11.8037 | 1 | 1 | 1 | 1 | 1 | 1 | 1 | 1 | 1 | 0 | 4 | 7 | 5 | 9 |
| 3 | Africa | -14.4524 | 14.4974 | 1 | 0 | 4 | 11 | 0 | 45 | 80 | 18 | 84 | 63 | 78 | 47 | 0 | 117 |
| 4 | Africa | -9.6966 | 9.9456 | 4 | 13 | 0 | 8 | 3 | 3 | 18 | 13 | 13 | 43 | 0 | 19 | 11 | 0 |
| 5 | Africa | -9.4295 | 6.4281 | 0 | 1 | 0 | 6 | 13 | 0 | 12 | 0 | 11 | 32 | 0 | 30 | 38 | 6 |
| 6 | Africa | -7.0926 | 31.7917 | 66 | 63 | 70 | 103 | 45 | 62 | 103 | 80 | 65 | 91 | 121 | 101 | 120 | 52 |
| 7 | Africa | -5.5471 | 7.54 | 2 | 17 | 17 | 13 | 72 | 49 | 18 | 59 | 91 | 135 | 156 | 180 | 65 | 88 |
| 8 | Africa | -3.99617 | 17.57069 | 3 | 3 | 3 | 3 | 3 | 3 | 3 | 3 | 3 | 0 | 4 | 16 | 60 | 0 |
| 9 | Africa | -1.5616 | 12.2383 | 22 | 11 | 0 | 0 | 93 | 84 | 159 | 142 | 97 | 149 | 60 | 136 | 129 | 70 |
| 10 | Africa | -1.0232 | 7.9465 | 27 | 23 | 26 | 29 | 39 | 17 | 37 | 76 | 96 | 133 | 191 | 159 | 148 | 138 |
| 11 | Africa | 0.8248 | 8.6195 | 11 | 9 | 28 | 3 | 0 | 37 | 0 | 0 | 0 | 0 | 3 | 5 | 0 | 72 |
| 12 | Africa | 1.6596 | 28.0339 | 84 | 23 | 53 | 10 | 117 | 7 | 94 | 70 | 20 | 43 | 63 | 65 | 12 | 68 |
| 13 | Africa | 2.3158 | 9.3077 | 2 | 4 | 0 | 9 | 11 | 3 | 22 | 0 | 14 | 67 | 11 | 37 | 50 | 14 |
| 14 | Africa | 8.0817 | 17.6078 | 2 | 2 | 2 | 2 | 1 | 3 | 0 | 10 | 6 | 4 | 17 | 23 | 24 | 7 |
| 15 | Africa | 8.6753 | 9.082 | 6 | 6 | 0 | 7 | 0 | 16 | 57 | 55 | 74 | 0 | 53 | 68 | 1 | 176 |
| 16 | Africa | 9 | 34 | 0 | 337 | 0 | 0 | 0 | 0 | 0 | 0 | 0 | 0 | 0 | 35 | 0 | 0 |
| 17 | Africa | 10 | 1.5 | 4 | 0 | 11 | 10 | 4 | 15 | 11 | 16 | 35 | 7 | 24 | 50 | 52 | 30 |
| 18 | Africa | 10.9408 | 21.0079 | 0 | 5 | 3 | 0 | 10 | 7 | 5 | 20 | 4 | 12 | 7 | 7 | 27 | 17 |
| 19 | Africa | 11.5021 | 3.848 | 21 | 1 | 21 | 0 | 46 | 3 | 0 | 45 | 2 | 56 | 75 | 0 | 23 | 42 |
| 20 | Africa | 11.6094 | -0.8037 | 0 | 6 | 6 | 0 | 6 | 10 | 14 | 58 | 17 | 18 | 33 | 4 | 32 | 19 |
| 21 | Africa | 17.22833 | 26.3351 | 4 | 4 | 4 | 4 | 4 | 4 | 4 | 4 | 4 | 5 | 8 | 5 | 7 | 0 |
| 22 | Africa | 17.8739 | -11.2027 | 4 | 4 | 4 | 4 | 3 | 6 | 6 | 2 | 13 | 0 | 2 | 10 | 5 | 9 |
| 23 | Africa | 18.4904 | -22.9576 | 27 | 7 | 4 | 3 | 2 | 0 | 0 | 5 | 0 | 27 | 34 | 4 | 76 | 25 |
| 24 | Africa | 18.7322 | 15.4542 | 3 | 3 | 3 | 2 | 4 | 0 | 6 | 4 | 0 | 4 | 14 | 7 | 18 | 12 |
| 25 | Africa | 20.9394 | 6.6111 | 0 | 0 | 0 | 0 | 0 | 0 | 7 | 0 | 13 | 5 | 0 | 24 | 0 | 13 |
| 26 | Africa | 21.7587 | -4.0383 | 1 | 7 | 3 | 0 | 68 | 12 | 38 | 60 | 0 | 69 | 49 | 71 | 65 | 0 |
| 27 | Africa | 22.9375 | -30.5595 | 139 | 146 | 89 | 687 | 315 | 338 | 98 | 317 | 581 | 730 | 555 | 362 | 356 | 315 |
| 28 | Africa | 28.2833 | -15.4167 | 4 | 4 | 0 | 5 | 12 | 9 | 6 | 6 | 4 | 0 | 46 | 5 | 0 | 0 |
| 29 | Africa | 29.8739 | -1.9403 | 1 | 0 | 7 | 35 | 0 | 9 | 5 | 60 | 50 | 20 | 129 | 69 | 60 | 84 |
| 30 | Africa | 30 | -20 | 4 | 4 | 4 | 4 | 3 | 4 | 12 | 16 | 12 | 7 | 8 | 10 | 15 | 74 |
| 31 | Africa | 30 | 26 | 90 | 11 | 0 | 16 | 15 | 87 | 5 | 21 | 0 | 5 | 0 | 0 | 0 | 0 |
| 32 | Africa | 30.2176 | 12.8628 | 8 | 5 | 0 | 3 | 7 | 8 | 21 | 9 | 1 | 32 | 5 | 24 | 22 | 3 |
| 33 | Africa | 31.4659 | -26.5225 | 5 | 7 | 7 | 0 | 2 | 6 | 0 | 18 | 9 | 11 | 39 | 0 | 0 | 0 |
| 34 | Africa | 32 | 1 | 4 | 4 | 4 | 4 | 4 | 3 | 5 | 0 | 0 | 0 | 0 | 0 | 0 | 0 |
| 35 | Africa | 34.8888 | -6.369 | 2 | 4 | 1 | 8 | 0 | 11 | 2 | 4 | 0 | 27 | 7 | 6 | 62 | 17 |
| 36 | Africa | 35.52956 | -18.6657 | 3 | 3 | 3 | 3 | 3 | 3 | 2 | 4 | 1 | 1 | 3 | 11 | 8 | 14 |
| 37 | Africa | 35.5296 | -18.6657 | 3 | 3 | 3 | 3 | 3 | 3 | 3 | 3 | 3 | 3 | 3 | 3 | 3 | 3 |
| 38 | Africa | 37.9062 | -0.0236 | 11 | 13 | 6 | 2 | 13 | 2 | 4 | 9 | 29 | 56 | 0 | 36 | 58 | 3 |
| 39 | Africa | 40.4897 | 9.145 | 11 | 2 | 14 | 13 | 3 | 15 | 47 | 13 | 9 | 34 | 11 | 34 | 34 | 33 |
| 40 | Africa | 42.5903 | 11.8251 | 3 | 3 | 2 | 4 | 5 | 7 | 1 | 0 | 3 | 0 | 0 | 25 | 3 | 0 |
| 41 | Africa | 45.1662 | -12.8275 | 5 | 6 | 0 | 5 | 14 | 10 | 0 | 5 | 47 | 0 | 62 | 28 | 0 | 59 |
| 42 | Africa | 46.1996 | 5.1521 | 3 | 5 | 6 | 8 | 4 | 1 | 3 | 7 | 4 | 3 | 17 | 10 | 37 | 10 |
| 43 | Africa | 46.8691 | -18.7669 | 4 | 4 | 4 | 4 | 0 | 3 | 1 | 0 | 11 | 0 | 0 | 0 | 0 | 60 |
| 44 | Africa | 55.2471 | -21.1351 | 21 | 26 | 46 | 5 | 81 | 69 | 98 | 167 | 180 | 150 | 101 | 153 | 173 | 121 |
| 45 | Africa | 55.492 | -4.6796 | 11 | 13 | 11 | 0 | 13 | 28 | 6 | 28 | 22 | 23 | 70 | 20 | 43 | 40 |
| 46 | Africa | 57.5 | -20.2 | 7 | 7 | 0 | 7 | 0 | 0 | 0 | 11 | 0 | 10 | 42 | 47 | 4 | 80 |
| 47 | Asia | 33.4299 | 35.1264 | 0 | 16 | 41 | 48 | 19 | 30 | 2 | 34 | 0 | 72 | 11 | 0 | 0 | 1 |
| 48 | Asia | 35 | 31 | 320 | 497 | 814 | 182 | 820 | 2041 | 1624 | 1771 | 2561 | 2076 | 2738 | 746 | 1291 | 1145 |
| 49 | Asia | 35.2332 | 31.9522 | 45 | 9 | 9 | 8 | 52 | 57 | 32 | 17 | 52 | 47 | 0 | 45 | 88 | 34 |
| 50 | Asia | 35.2433 | 38.9637 | 1108 | 1410 | 788 | 0 | 0 | 0 | 0 | 662 | 372 | 160 | 390 | 678 | 1374 | 684 |
| 51 | Asia | 35.8623 | 33.8547 | 0 | 30 | 0 | 24 | 0 | 11 | 0 | 2 | 0 | 0 | 58 | 0 | 0 | 7 |
| 52 | Asia | 36.51 | 31.24 | 0 | 32 | 23 | 11 | 21 | 0 | 11 | 2 | 29 | 24 | 38 | 27 | 11 | 17 |
| 53 | Asia | 38.9968 | 34.8021 | 2 | 2 | 2 | 2 | 2 | 2 | 2 | 2 | 2 | 2 | 2 | 2 | 2 | 2 |
| 54 | Asia | 38.99682 | 34.80208 | 2 | 2 | 2 | 2 | 2 | 2 | 2 | 3 | 3 | 3 | 7 | 12 | 0 | 1 |
| 55 | Asia | 44 | 33 | 0 | 4 | 8 | 0 | 0 | 0 | 23 | 3 | 0 | 0 | 11 | 0 | 18 | 13 |
| 56 | Asia | 45 | 24 | 233 | 68 | 472 | 274 | 312 | 362 | 459 | 374 | 361 | 347 | 343 | 395 | 350 | 273 |
| 57 | Asia | 45.0382 | 40.0691 | 23 | 61 | 29 | 73 | 27 | 74 | 69 | 76 | 36 | 49 | 70 | 72 | 70 | 36 |
| 58 | Asia | 47.5769 | 40.1431 | 23 | 41 | 11 | 3 | 0 | 0 | 21 | 32 | 35 | 0 | 24 | 37 | 21 | 0 |
| 59 | Asia | 47.75 | 29.5 | 0 | 14 | 0 | 45 | 43 | 48 | 0 | 33 | 0 | 0 | 52 | 16 | 0 | 19 |
| 60 | Asia | 50.55 | 26.0275 | 56 | 0 | 42 | 46 | 0 | 0 | 13 | 10 | 0 | 0 | 9 | 7 | 0 | 7 |
| 61 | Asia | 51.1839 | 25.3548 | 304 | 133 | 87 | 62 | 214 | 152 | 151 | 66 | 200 | 36 | 135 | 86 | 252 | 118 |
| 62 | Asia | 53 | 32 | 2901 | 2635 | 2149 | 1997 | 3361 | 3343 | 3354 | 0 | 0 | 334 | 686 | 593 | 756 | 792 |
| 63 | Asia | 54 | 24 | 206 | 70 | 217 | 93 | 69 | 94 | 239 | 22 | 160 | 343 | 331 | 172 | 353 | 362 |
| 64 | Asia | 57 | 21 | 7 | 16 | 31 | 43 | 0 | 66 | 23 | 18 | 113 | 45 | 82 | 126 | 48 | 9 |
| 65 | Asia | 64.5853 | 41.3775 | 0 | 13 | 29 | 9 | 35 | 35 | 2 | 17 | 12 | 23 | 26 | 58 | 24 | 24 |
| **Row** | **Continents** | **Longitude** | **Latitude** | **30 Mar** | |  |  |  |  | **6 Apr** | | |  |  |  | **12 Apr** | |
| 66 | Asia | 65 | 33 | 23 | 23 | 21 | 0 | 1 | 0 | 39 | 7 | 58 | 0 | 4 | 14 | 0 | 17 |
| 67 | Asia | 66.9237 | 48.0196 | 74 | 28 | 61 | 43 | 75 | 99 | 130 | 82 | 69 | 67 | 57 | 35 | 82 | 55 |
| 68 | Asia | 69.3451 | 30.3753 | 55 | 306 | 283 | 527 | 124 | 286 | 243 | 268 | 428 | 319 | 412 | 366 | 389 | 404 |
| 69 | Asia | 73.2207 | 3.2028 | 14 | 51 | 16 | 9 | 47 | 18 | 57 | 43 | 27 | 47 | 27 | 14 | 0 | 2 |
| 70 | Asia | 74.7661 | 41.2044 | 10 | 10 | 6 | 10 | 5 | 0 | 10 | 0 | 0 | 23 | 18 | 10 | 0 | 0 |
| 71 | Asia | 78 | 21 | 98 | 65 | 14 | 122 | 69 | 100 | 147 | 99 | 45 | 55 | 129 | 69 | 107 | 77 |
| 72 | Asia | 81 | 7 | 32 | 63 | 24 | 8 | 65 | 13 | 51 | 104 | 93 | 86 | 139 | 58 | 97 | 32 |
| 73 | Asia | 84.25 | 28.1667 | 0 | 0 | 0 | 0 | 0 | 0 | 0 | 0 | 0 | 0 | 0 | 0 | 0 | 0 |
| 74 | Asia | 85.2401 | 41.1129 | 0 | 0 | 0 | 0 | 0 | 0 | 0 | 0 | 0 | 0 | 0 | 0 | 0 | 0 |
| 75 | Asia | 88.0924 | 31.6927 | 5 | 5 | 5 | 5 | 5 | 5 | 5 | 5 | 5 | 5 | 5 | 5 | 5 | 5 |
| 76 | Asia | 90.3563 | 23.685 | 0 | 9 | 8 | 32 | 38 | 20 | 56 | 27 | 36 | 32 | 27 | 15 | 3 | 16 |
| 77 | Asia | 90.4336 | 27.5142 | 1 | 1 | 4 | 1 | 1 | 2 | 0 | 0 | 1 | 0 | 0 | 4 | 6 | 0 |
| 78 | Asia | 95.956 | 21.9162 | 2 | 2 | 2 | 2 | 2 | 2 | 2 | 2 | 2 | 2 | 2 | 0 | 0 | 0 |
| 79 | Asia | 95.9956 | 35.7452 | 1 | 1 | 1 | 1 | 1 | 1 | 1 | 1 | 1 | 1 | 1 | 1 | 1 | 1 |
| 80 | Asia | 101 | 15 | 166 | 139 | 126 | 130 | 158 | 142 | 194 | 191 | 172 | 125 | 116 | 180 | 168 | 143 |
| 81 | Asia | 101.0583 | 37.8099 | 19 | 0 | 4 | 8 | 17 | 11 | 19 | 5 | 28 | 22 | 0 | 12 | 11 | 28 |
| 82 | Asia | 101.487 | 24.974 | 0 | 3 | 6 | 1 | 5 | 0 | 1 | 6 | 2 | 1 | 6 | 3 | 17 | 4 |
| 83 | Asia | 102.4955 | 19.85627 | 2 | 2 | 2 | 2 | 2 | 2 | 2 | 2 | 0 | 5 | 10 | 20 | 11 | 7 |
| 84 | Asia | 102.7103 | 30.6171 | 1 | 16 | 19 | 0 | 0 | 4 | 6 | 5 | 0 | 0 | 21 | 20 | 14 | 11 |
| 85 | Asia | 103.8333 | 1.2833 | 97 | 106 | 82 | 76 | 33 | 37 | 42 | 66 | 119 | 72 | 108 | 37 | 133 | 125 |
| 86 | Asia | 103.8467 | 46.8625 | 4 | 2 | 25 | 9 | 0 | 14 | 17 | 0 | 17 | 18 | 14 | 3 | 6 | 11 |
| 87 | Asia | 104.9167 | 11.55 | 28 | 22 | 30 | 60 | 157 | 53 | 154 | 162 | 47 | 108 | 118 | 113 | 70 | 108 |
| 88 | Asia | 106.1655 | 37.2692 | 2 | 0 | 0 | 0 | 0 | 0 | 0 | 0 | 0 | 0 | 0 | 0 | 0 | 0 |
| 89 | Asia | 106.8748 | 26.8154 | 2 | 1 | 0 | 0 | 0 | 2 | 0 | 0 | 5 | 2 | 1 | 1 | 2 | 5 |
| 90 | Asia | 107.874 | 30.0572 | 2 | 2 | 2 | 2 | 2 | 2 | 1 | 3 | 3 | 0 | 0 | 0 | 0 | 4 |
| 91 | Asia | 108 | 16 | 42 | 73 | 0 | 94 | 92 | 51 | 136 | 38 | 88 | 118 | 30 | 0 | 59 | 11 |
| 92 | Asia | 108.7881 | 23.8298 | 3 | 2 | 1 | 0 | 0 | 1 | 0 | 0 | 6 | 0 | 3 | 6 | 3 | 5 |
| 93 | Asia | 108.8701 | 35.1917 | 0 | 0 | 0 | 0 | 0 | 1 | 0 | 0 | 1 | 2 | 0 | 4 | 3 | 0 |
| 94 | Asia | 109.7453 | 19.1959 | 3 | 3 | 3 | 3 | 3 | 3 | 3 | 3 | 3 | 3 | 3 | 3 | 3 | 3 |
| 95 | Asia | 111.7088 | 27.6104 | 2 | 1 | 2 | 1 | 2 | 2 | 2 | 2 | 2 | 2 | 2 | 2 | 2 | 2 |
| 96 | Asia | 112.2707 | 30.9756 | 376 | 0 | 93 | 0 | 0 | 53 | 131 | 0 | 393 | 604 | 107 | 226 | 431 | 167 |
| 97 | Asia | 112.2922 | 37.5777 | 0 | 0 | 0 | 0 | 0 | 0 | 0 | 0 | 0 | 0 | 0 | 0 | 0 | 0 |
| 98 | Asia | 112.5 | 2.5 | 364 | 239 | 393 | 468 | 344 | 322 | 311 | 370 | 284 | 285 | 361 | 229 | 382 | 276 |
| 99 | Asia | 113.4244 | 23.3417 | 11 | 32 | 22 | 49 | 66 | 20 | 55 | 12 | 69 | 78 | 62 | 72 | 58 | 22 |
| 100 | Asia | 113.55 | 22.1667 | 2 | 3 | 1 | 2 | 24 | 21 | 3 | 29 | 14 | 12 | 48 | 38 | 50 | 60 |
| 101 | Asia | 113.614 | 33.882 | 0 | 0 | 5 | 2 | 1 | 2 | 1 | 3 | 3 | 0 | 1 | 0 | 1 | 2 |
| 102 | Asia | 113.9213 | -0.7893 | 119 | 186 | 187 | 179 | 211 | 92 | 244 | 173 | 168 | 126 | 182 | 164 | 144 | 153 |
| 103 | Asia | 114.2 | 22.3 | 71 | 33 | 36 | 58 | 12 | 32 | 65 | 56 | 35 | 56 | 26 | 91 | 34 | 48 |
| 104 | Asia | 114.7277 | 4.5353 | 42 | 0 | 89 | 60 | 64 | 47 | 76 | 71 | 62 | 85 | 101 | 174 | 70 | 53 |
| 105 | Asia | 115.7221 | 27.614 | 2 | 2 | 2 | 2 | 2 | 2 | 1 | 1 | 0 | 0 | 2 | 0 | 1 | 4 |
| 106 | Asia | 116.1306 | 39.549 | 7 | 24 | 7 | 7 | 7 | 7 | 6 | 6 | 4 | 6 | 8 | 4 | 6 | 10 |
| 107 | Asia | 116.4142 | 40.1824 | 36 | 43 | 37 | 19 | 57 | 0 | 56 | 27 | 22 | 26 | 3 | 14 | 9 | 40 |
| 108 | Asia | 117.2264 | 31.8257 | 10 | 10 | 9 | 10 | 10 | 10 | 10 | 10 | 10 | 10 | 10 | 10 | 10 | 10 |
| 109 | Asia | 117.323 | 39.3054 | 7 | 7 | 7 | 6 | 6 | 4 | 6 | 6 | 10 | 20 | 5 | 16 | 36 | 13 |
| 110 | Asia | 117.9874 | 26.0789 | 2 | 2 | 2 | 2 | 0 | 0 | 18 | 0 | 16 | 0 | 16 | 24 | 4 | 28 |
| 111 | Asia | 118.1498 | 36.3427 | 0 | 0 | 32 | 74 | 89 | 177 | 159 | 171 | 200 | 50 | 205 | 201 | 56 | 166 |
| 112 | Asia | 119.455 | 32.9711 | 1 | 1 | 1 | 1 | 1 | 1 | 0 | 2 | 3 | 0 | 26 | 24 | 18 | 10 |
| 113 | Asia | 120.0934 | 29.1832 | 8 | 10 | 0 | 0 | 3 | 0 | 19 | 12 | 0 | 9 | 27 | 27 | 28 | 73 |
| 114 | Asia | 121 | 23.7 | 22 | 80 | 55 | 9 | 10 | 3 | 13 | 69 | 20 | 0 | 38 | 33 | 40 | 7 |
| 115 | Asia | 122 | 13 | 113 | 106 | 349 | 110 | 0 | 298 | 307 | 240 | 226 | 288 | 175 | 386 | 256 | 195 |
| 116 | Asia | 122.6085 | 41.2956 | 1 | 13 | 0 | 1 | 0 | 0 | 6 | 0 | 0 | 0 | 0 | 0 | 0 | 0 |
| 117 | Asia | 125.7275 | -8.8742 | 0 | 0 | 0 | 0 | 0 | 0 | 0 | 0 | 0 | 0 | 0 | 0 | 0 | 0 |
| 118 | Asia | 125.7275 | -8.87422 | 0 | 0 | 0 | 0 | 0 | 0 | 0 | 0 | 0 | 4 | 2 | 0 | 0 | 3 |
| 119 | Asia | 126.1923 | 43.6661 | 1 | 1 | 1 | 1 | 1 | 1 | 1 | 1 | 1 | 0 | 1 | 0 | 0 | 1 |
| 120 | Asia | 127.7615 | 47.862 | 0 | 5 | 7 | 15 | 1 | 0 | 0 | 5 | 0 | 0 | 7 | 18 | 14 | 3 |
| 121 | Asia | 128 | 36 | 0 | 0 | 180 | 1126 | 0 | 1286 | 1442 | 927 | 700 | 451 | 0 | 0 | 913 | 0 |
| 122 | Asia | 138 | 36 | 607 | 441 | 517 | 169 | 0 | 173 | 288 | 411 | 593 | 0 | 70 | 0 | 551 | 817 |
| 123 | Australian | 115.8605 | -31.9505 | 53 | 79 | 61 | 128 | 74 | 67 | 182 | 198 | 121 | 163 | 205 | 176 | 132 | 102 |
| 124 | Australian | 130.8456 | -12.4634 | 23 | 3 | 0 | 4 | 14 | 3 | 39 | 4 | 0 | 6 | 0 | 9 | 0 | 0 |
| 125 | Australian | 138.6007 | -34.9285 | 40 | 13 | 49 | 37 | 80 | 66 | 155 | 58 | 155 | 57 | 134 | 158 | 117 | 159 |
| 126 | Australian | 143.9555 | -6.315 | 0 | 0 | 0 | 0 | 0 | 0 | 0 | 4 | 2 | 0 | 0 | 3 | 42 | 4 |
| 127 | Australian | 144.9631 | -37.8136 | 224 | 95 | 125 | 137 | 109 | 92 | 274 | 110 | 91 | 172 | 94 | 92 | 90 | 63 |
| 128 | Australian | 145.9707 | -41.4545 | 0 | 38 | 20 | 71 | 77 | 28 | 26 | 64 | 58 | 88 | 0 | 40 | 73 | 40 |
| 129 | Australian | 149.0124 | -35.4735 | 0 | 10 | 0 | 2 | 20 | 38 | 33 | 0 | 13 | 114 | 89 | 92 | 210 | 157 |
| 130 | Australian | 151.2093 | -33.8688 | 260 | 336 | 38 | 452 | 245 | 500 | 333 | 342 | 254 | 579 | 345 | 431 | 362 | 374 |
| 131 | Australian | 153.4 | -28.0167 | 116 | 126 | 37 | 135 | 157 | 74 | 125 | 168 | 178 | 156 | 177 | 164 | 170 | 103 |
| 132 | Australian | 165.618 | -20.9043 | 3 | 3 | 3 | 0 | 4 | 4 | 10 | 0 | 0 | 0 | 30 | 0 | 26 | 32 |
| 133 | Australian | 174.886 | -40.9006 | 83 | 19 | 5 | 15 | 0 | 0 | 49 | 67 | 185 | 109 | 182 | 221 | 140 | 151 |
| 134 | Australian | 178.065 | -17.7134 | 0 | 0 | 0 | 0 | 0 | 0 | 0 | 0 | 0 | 0 | 14 | 44 | 28 | 14 |
| **Row** | **Continents** | **Longitude** | **Latitude** | **30 Mar** | |  |  |  |  | **6 Apr** | | |  |  |  | **12 Apr** | |
| 135 | Europe | -61.0242 | 14.6415 | 0 | 0 | 0 | 30 | 18 | 3 | 13 | 37 | 53 | 20 | 6 | 15 | 69 | 65 |
| 136 | Europe | -53.1258 | 3.9339 | 7 | 22 | 9 | 28 | 20 | 23 | 11 | 24 | 17 | 72 | 39 | 56 | 27 | 43 |
| 137 | Europe | -19.0208 | 64.9631 | 30 | 69 | 66 | 98 | 78 | 84 | 66 | 65 | 52 | 124 | 70 | 132 | 118 | 106 |
| 138 | Europe | -8.2245 | 39.3999 | 685 | 580 | 440 | 631 | 715 | 663 | 736 | 295 | 449 | 469 | 638 | 616 | 607 | 506 |
| 139 | Europe | -7.6921 | 53.1424 | 246 | 189 | 503 | 417 | 383 | 404 | 431 | 439 | 370 | 426 | 527 | 353 | 435 | 425 |
| 140 | Europe | -6.9118 | 61.8926 | 9 | 53 | 66 | 47 | 60 | 36 | 123 | 83 | 63 | 91 | 47 | 96 | 120 | 130 |
| 141 | Europe | -5.3536 | 36.1408 | 5 | 0 | 0 | 21 | 0 | 26 | 18 | 15 | 11 | 2 | 70 | 65 | 0 | 19 |
| 142 | Europe | -4.5481 | 54.2361 | 10 | 10 | 10 | 10 | 7 | 23 | 24 | 13 | 84 | 11 | 16 | 6 | 87 | 19 |
| 143 | Europe | -4 | 40 | 6727 | 6240 | 5585 | 6288 | 6138 | 6086 | 6011 | 7267 | 6926 | 7347 | 6739 | 6679 | 6742 | 6721 |
| 144 | Europe | -3.436 | 55.3781 | 4259 | 5602 | 2810 | 3731 | 5831 | 6382 | 1712 | 5209 | 4958 | 5358 | 5382 | 5797 | 5279 | 4990 |
| 145 | Europe | 1.5218 | 42.5063 | 17 | 32 | 1 | 0 | 51 | 87 | 64 | 57 | 60 | 47 | 76 | 44 | 82 | 32 |
| 146 | Europe | 2.2137 | 46.2276 | 5006 | 6917 | 5871 | 6641 | 6828 | 5938 | 5957 | 5500 | 6595 | 6372 | 5558 | 5257 | 6864 | 6719 |
| 147 | Europe | 4 | 50.8333 | 1855 | 1776 | 1727 | 1799 | 4313 | 2403 | 5472 | 2476 | 2301 | 5448 | 5695 | 5763 | 6119 | 6005 |
| 148 | Europe | 5.2913 | 52.1326 | 1192 | 1190 | 785 | 1001 | 361 | 3932 | 1922 | 1718 | 4838 | 5810 | 5439 | 5579 | 5367 | 5362 |
| 149 | Europe | 6.1296 | 49.8153 | 150 | 248 | 529 | 531 | 474 | 576 | 565 | 344 | 470 | 302 | 437 | 699 | 330 | 421 |
| 150 | Europe | 7.4167 | 43.7333 | 3 | 9 | 22 | 7 | 33 | 26 | 2 | 0 | 7 | 0 | 0 | 0 | 44 | 0 |
| 151 | Europe | 8.2275 | 46.8182 | 420 | 435 | 1733 | 4048 | 6283 | 5265 | 5235 | 6240 | 5532 | 6009 | 5330 | 4426 | 5604 | 5116 |
| 152 | Europe | 8.4689 | 60.472 | 369 | 436 | 642 | 332 | 597 | 491 | 516 | 322 | 324 | 312 | 365 | 310 | 378 | 311 |
| 153 | Europe | 9 | 51 | 5511 | 5389 | 4985 | 6818 | 8786 | 7304 | 6572 | 7011 | 7114 | 7150 | 7142 | 7158 | 7154 | 7107 |
| 154 | Europe | 9.5018 | 56.2639 | 133 | 166 | 169 | 75 | 117 | 203 | 220 | 120 | 158 | 171 | 178 | 209 | 190 | 190 |
| 155 | Europe | 9.55 | 47.14 | 10 | 0 | 0 | 17 | 0 | 0 | 0 | 0 | 0 | 0 | 15 | 55 | 8 | 33 |
| 156 | Europe | 12 | 43 | 7274 | 5661 | 7353 | 7361 | 7233 | 7354 | 7339 | 7271 | 7239 | 7265 | 7282 | 7222 | 7222 | 7222 |
| 157 | Europe | 12.4534 | 41.9029 | 0 | 0 | 0 | 0 | 0 | 0 | 0 | 0 | 0 | 0 | 3 | 0 | 0 | 0 |
| 158 | Europe | 12.4578 | 43.9424 | 4 | 54 | 59 | 89 | 1 | 0 | 0 | 0 | 77 | 0 | 25 | 0 | 0 | 80 |
| 159 | Europe | 14.3754 | 35.9375 | 13 | 0 | 7 | 21 | 0 | 14 | 41 | 0 | 37 | 40 | 0 | 17 | 23 | 31 |
| 160 | Europe | 14.5501 | 47.5162 | 1968 | 0 | 2268 | 947 | 1339 | 1216 | 357 | 841 | 175 | 633 | 503 | 164 | 181 | 207 |
| 161 | Europe | 14.9955 | 46.1512 | 0 | 55 | 26 | 123 | 56 | 66 | 26 | 53 | 43 | 14 | 32 | 37 | 91 | 28 |
| 162 | Europe | 15.2 | 45.1 | 4 | 17 | 64 | 27 | 24 | 37 | 58 | 0 | 111 | 69 | 10 | 82 | 106 | 98 |
| 163 | Europe | 15.473 | 49.8175 | 335 | 434 | 415 | 548 | 474 | 596 | 605 | 332 | 458 | 328 | 653 | 630 | 265 | 407 |
| 164 | Europe | 16 | 63 | 283 | 385 | 368 | 430 | 527 | 521 | 457 | 229 | 254 | 484 | 344 | 488 | 367 | 300 |
| 165 | Europe | 17.6791 | 43.9159 | 38 | 51 | 35 | 55 | 34 | 95 | 90 | 51 | 56 | 48 | 65 | 95 | 65 | 22 |
| 166 | Europe | 19.1451 | 51.9194 | 168 | 101 | 408 | 196 | 467 | 433 | 392 | 455 | 347 | 444 | 410 | 444 | 277 | 336 |
| 167 | Europe | 19.3 | 42.5 | 3 | 0 | 5 | 15 | 2 | 0 | 0 | 0 | 10 | 14 | 18 | 0 | 11 | 34 |
| 168 | Europe | 19.5033 | 47.1625 | 44 | 45 | 67 | 47 | 37 | 66 | 92 | 61 | 0 | 101 | 11 | 36 | 3 | 65 |
| 169 | Europe | 19.699 | 48.669 | 35 | 13 | 34 | 40 | 85 | 41 | 105 | 71 | 111 | 0 | 91 | 9 | 17 | 27 |
| 170 | Europe | 20.1683 | 41.1533 | 13 | 36 | 37 | 49 | 33 | 48 | 45 | 51 | 26 | 91 | 38 | 66 | 62 | 19 |
| 171 | Europe | 20.90298 | 42.60264 | 10 | 10 | 10 | 10 | 10 | 10 | 10 | 10 | 10 | 10 | 0 | 77 | 0 | 30 |
| 172 | Europe | 21.0059 | 44.0165 | 113 | 172 | 296 | 162 | 288 | 93 | 244 | 333 | 224 | 291 | 358 | 259 | 434 | 143 |
| 173 | Europe | 21.7453 | 41.6086 | 3 | 0 | 12 | 19 | 31 | 22 | 20 | 0 | 57 | 15 | 13 | 29 | 27 | 11 |
| 174 | Europe | 21.8243 | 39.0742 | 25 | 0 | 32 | 109 | 6 | 108 | 100 | 27 | 59 | 42 | 88 | 73 | 62 | 121 |
| 175 | Europe | 23.8813 | 55.1694 | 37 | 27 | 70 | 63 | 48 | 47 | 60 | 73 | 48 | 88 | 57 | 40 | 122 | 75 |
| 176 | Europe | 24.6032 | 56.8796 | 47 | 19 | 56 | 34 | 24 | 0 | 64 | 25 | 2 | 61 | 48 | 13 | 0 | 0 |
| 177 | Europe | 24.9668 | 45.9432 | 270 | 316 | 267 | 442 | 329 | 313 | 522 | 490 | 518 | 470 | 408 | 432 | 386 | 581 |
| 178 | Europe | 25.0136 | 58.5953 | 1 | 0 | 80 | 53 | 73 | 43 | 49 | 59 | 38 | 40 | 64 | 58 | 108 | 72 |
| 179 | Europe | 25.4858 | 42.7339 | 53 | 37 | 13 | 33 | 95 | 75 | 85 | 58 | 56 | 52 | 91 | 35 | 17 | 60 |
| 180 | Europe | 26 | 64 | 62 | 142 | 100 | 55 | 96 | 74 | 194 | 132 | 170 | 106 | 170 | 175 | 101 | 107 |
| 181 | Europe | 27.9534 | 53.7098 | 20 | 0 | 29 | 22 | 7 | 1 | 95 | 0 | 60 | 0 | 48 | 9 | 0 | 52 |
| 182 | Europe | 28.3699 | 47.4116 | 31 | 29 | 0 | 17 | 25 | 19 | 76 | 47 | 32 | 18 | 38 | 0 | 85 | 0 |
| 183 | Europe | 31.1656 | 48.3794 | 70 | 76 | 88 | 70 | 12 | 57 | 18 | 88 | 39 | 19 | 71 | 67 | 66 | 87 |
| 184 | Europe | 43.3569 | 42.3154 | 2 | 6 | 0 | 23 | 0 | 1 | 11 | 38 | 0 | 4 | 14 | 11 | 4 | 32 |
| 185 | Europe | 90 | 60 | 283 | 384 | 360 | 417 | 638 | 649 | 527 | 532 | 553 | 538 | 594 | 494 | 558 | 303 |
| 186 | North America | -135 | 64.2823 | 0 | 0 | 0 | 0 | 0 | 0 | 0 | 0 | 0 | 0 | 79 | 11 | 1 | 11 |
| 187 | North America | -124.846 | 64.8255 | 0 | 0 | 0 | 0 | 0 | 0 | 0 | 0 | 0 | 0 | 5 | 1 | 5 | 0 |
| 188 | North America | -123.121 | 49.2827 | 23 | 145 | 26 | 88 | 70 | 22 | 124 | 0 | 146 | 104 | 27 | 175 | 0 | 158 |
| 189 | North America | -122.666 | 37.6489 | 0 | 0 | 17 | 0 | 40 | 0 | 52 | 0 | 31 | 15 | 38 | 77 | 6 | 47 |
| 190 | North America | -116.577 | 53.9333 | 34 | 124 | 33 | 96 | 109 | 93 | 183 | 91 | 91 | 110 | 66 | 101 | 18 | 88 |
| 191 | North America | -106.451 | 52.9399 | 0 | 175 | 0 | 0 | 90 | 0 | 312 | 10 | 0 | 339 | 161 | 261 | 215 | 262 |
| 192 | North America | -106.347 | 56.1304 | 0 | 0 | 0 | 0 | 0 | 0 | 0 | 0 | 0 | 0 | 0 | 0 | 0 | 0 |
| 193 | North America | -102.553 | 23.6345 | 30 | 166 | 96 | 84 | 119 | 111 | 159 | 183 | 124 | 145 | 173 | 111 | 167 | 196 |
| 194 | North America | -98.8139 | 53.7609 | 54 | 24 | 0 | 16 | 0 | 30 | 56 | 0 | 19 | 48 | 18 | 162 | 33 | 27 |
| 195 | North America | -95.7129 | 37.0902 | 18334 | 14984 | 18292 | 16432 | 18096 | 17287 | 16789 | 16808 | 17928 | 16538 | 17460 | 17651 | 16453 | 16628 |
| 196 | North America | -90.2308 | 15.7835 | 7 | 16 | 3 | 61 | 63 | 37 | 35 | 24 | 38 | 75 | 49 | 34 | 140 | 96 |
| 197 | North America | -88.8965 | 13.7942 | 2 | 2 | 2 | 1 | 13 | 27 | 19 | 23 | 19 | 73 | 45 | 0 | 58 | 17 |
| 198 | North America | -86.2419 | 15.2 | 58 | 23 | 35 | 75 | 33 | 13 | 37 | 109 | 86 | 93 | 100 | 107 | 173 | 253 |
| 199 | North America | -85.3232 | 51.2538 | 0 | 211 | 119 | 184 | 370 | 332 | 313 | 266 | 302 | 282 | 296 | 275 | 260 | 499 |
| 200 | North America | -85.2072 | 12.8654 | 0 | 0 | 0 | 0 | 10 | 3 | 15 | 14 | 0 | 21 | 1 | 11 | 36 | 35 |
| 201 | North America | -81.2546 | 19.3133 | 2 | 9 | 0 | 36 | 25 | 9 | 18 | 48 | 11 | 58 | 32 | 71 | 17 | 14 |
| 202 | North America | -80.7821 | 8.538 | 124 | 140 | 184 | 239 | 213 | 187 | 275 | 184 | 185 | 285 | 207 | 209 | 239 | 235 |
| 203 | North America | -80 | 22 | 20 | 8 | 69 | 67 | 90 | 98 | 86 | 103 | 135 | 135 | 164 | 134 | 198 | 181 |
| **Row** | **Continents** | **Longitude** | **Latitude** | **30 Mar** | |  |  |  |  | **6 Apr** | | |  |  |  | **12 Apr** | |
| 204 | North America | -77.3963 | 25.0343 | 1 | 12 | 14 | 19 | 15 | 25 | 28 | 48 | 2 | 55 | 53 | 40 | 62 | 22 |
| 205 | North America | -77.2975 | 18.1096 | 0 | 53 | 17 | 49 | 36 | 84 | 115 | 83 | 139 | 142 | 97 | 139 | 94 | 146 |
| 206 | North America | -73.5491 | 52.9399 | 397 | 549 | 444 | 706 | 0 | 379 | 428 | 413 | 398 | 327 | 683 | 291 | 590 | 368 |
| 207 | North America | -72.2852 | 18.9712 | 1 | 1 | 1 | 1 | 37 | 25 | 12 | 72 | 44 | 35 | 29 | 44 | 0 | 80 |
| 208 | North America | -71.7979 | 21.694 | 1 | 1 | 1 | 1 | 1 | 1 | 1 | 1 | 1 | 1 | 1 | 1 | 60 | 27 |
| 209 | North America | -70.1627 | 18.7357 | 40 | 93 | 103 | 88 | 193 | 199 | 198 | 241 | 173 | 238 | 282 | 180 | 283 | 273 |
| 210 | North America | -70.0358 | 12.5186 | 7 | 12 | 10 | 28 | 21 | 14 | 75 | 41 | 68 | 61 | 69 | 116 | 71 | 148 |
| 211 | North America | -66.4619 | 46.5653 | 15 | 40 | 50 | 40 | 38 | 81 | 35 | 23 | 0 | 72 | 48 | 112 | 68 | 107 |
| 212 | North America | -64.7505 | 32.3078 | 2 | 2 | 2 | 0 | 4 | 17 | 31 | 26 | 10 | 2 | 6 | 43 | 45 | 64 |
| 213 | North America | -64.64 | 18.4207 | 2 | 2 | 2 | 2 | 2 | 2 | 2 | 2 | 2 | 2 | 2 | 2 | 0 | 4 |
| 214 | North America | -63.7443 | 44.682 | 29 | 21 | 12 | 25 | 15 | 46 | 52 | 81 | 74 | 92 | 56 | 94 | 46 | 61 |
| 215 | North America | -63.4168 | 46.5107 | 5 | 6 | 5 | 13 | 0 | 18 | 5 | 21 | 3 | 18 | 15 | 26 | 22 | 50 |
| 216 | North America | -63.0686 | 18.2206 | 0 | 0 | 0 | 0 | 0 | 0 | 0 | 0 | 0 | 0 | 0 | 0 | 0 | 2 |
| 217 | North America | -63.0548 | 18.0425 | 0 | 0 | 0 | 0 | 0 | 1 | 1 | 0 | 2 | 0 | 19 | 20 | 0 | 20 |
| 218 | North America | -63.0501 | 18.0708 | 3 | 0 | 0 | 6 | 6 | 30 | 7 | 0 | 9 | 29 | 16 | 60 | 35 | 27 |
| 219 | North America | -62.8333 | 17.9 | 27 | 4 | 15 | 2 | 14 | 0 | 12 | 25 | 4 | 10 | 10 | 0 | 28 | 5 |
| 220 | North America | -62.783 | 17.35782 | 2 | 2 | 2 | 2 | 2 | 2 | 2 | 2 | 2 | 0 | 4 | 8 | 6 | 10 |
| 221 | North America | -62.1874 | 16.7425 | 2 | 2 | 1 | 3 | 3 | 5 | 12 | 0 | 14 | 5 | 4 | 10 | 10 | 0 |
| 222 | North America | -61.7964 | 17.0608 | 5 | 12 | 0 | 14 | 5 | 2 | 13 | 14 | 5 | 32 | 10 | 11 | 0 | 0 |
| 223 | North America | -61.679 | 12.1165 | 2 | 2 | 2 | 2 | 2 | 2 | 1 | 3 | 2 | 5 | 6 | 0 | 8 | 0 |
| 224 | North America | -61.2872 | 12.9843 | 2 | 5 | 12 | 0 | 14 | 5 | 2 | 13 | 10 | 4 | 26 | 2 | 2 | 2 |
| 225 | North America | -57.6604 | 53.1355 | 26 | 10 | 28 | 38 | 8 | 57 | 13 | 73 | 59 | 46 | 70 | 113 | 84 | 131 |
| 226 | North America | -42.6043 | 71.7069 | 7 | 0 | 3 | 2 | 0 | 0 | 0 | 0 | 30 | 22 | 21 | 13 | 61 | 23 |
| 227 | North America | 113.9448 | 44.0935 | 5 | 5 | 5 | 5 | 5 | 5 | 5 | 5 | 5 | 0 | 1 | 18 | 4 | 12 |
| 228 | North America | 121.4491 | 31.202 | 0 | 29 | 20 | 10 | 40 | 53 | 56 | 10 | 20 | 27 | 26 | 85 | 29 | 8 |
| 229 | South America | -83.7534 | 9.7489 | 23 | 130 | 104 | 123 | 250 | 195 | 213 | 244 | 207 | 191 | 188 | 182 | 150 | 177 |
| 230 | South America | -78.1834 | -1.8312 | 362 | 397 | 150 | 200 | 542 | 452 | 431 | 400 | 385 | 398 | 472 | 310 | 459 | 443 |
| 231 | South America | -75.0152 | -9.19 | 207 | 146 | 119 | 166 | 174 | 137 | 260 | 108 | 275 | 158 | 215 | 141 | 89 | 174 |
| 232 | South America | -74.2973 | 4.5709 | 128 | 128 | 230 | 180 | 197 | 111 | 266 | 198 | 231 | 229 | 174 | 244 | 255 | 180 |
| 233 | South America | -71.543 | -35.6751 | 242 | 503 | 205 | 374 | 442 | 455 | 403 | 636 | 707 | 626 | 548 | 602 | 433 | 561 |
| 234 | South America | -68.99 | 12.1696 | 8 | 7 | 6 | 11 | 19 | 14 | 0 | 13 | 40 | 21 | 86 | 53 | 15 | 61 |
| 235 | South America | -66.5897 | 6.4238 | 70 | 88 | 81 | 36 | 73 | 62 | 0 | 66 | 73 | 50 | 150 | 63 | 59 | 47 |
| 236 | South America | -63.6167 | -38.4161 | 157 | 78 | 25 | 273 | 164 | 151 | 157 | 205 | 183 | 114 | 152 | 176 | 162 | 156 |
| 237 | South America | -63.5887 | -16.2902 | 0 | 19 | 18 | 4 | 73 | 23 | 60 | 91 | 26 | 139 | 21 | 171 | 69 | 146 |
| 238 | South America | -61.5833 | 16.25 | 8 | 0 | 57 | 78 | 0 | 64 | 50 | 0 | 39 | 36 | 4 | 96 | 92 | 138 |
| 239 | South America | -61.371 | 15.415 | 2 | 2 | 2 | 2 | 2 | 2 | 1 | 4 | 9 | 5 | 18 | 11 | 5 | 7 |
| 240 | South America | -61.2225 | 10.6918 | 7 | 14 | 23 | 5 | 36 | 109 | 69 | 61 | 95 | 52 | 101 | 76 | 92 | 129 |
| 241 | South America | -60.9789 | 13.9094 | 7 | 4 | 13 | 0 | 1 | 12 | 3 | 0 | 20 | 11 | 31 | 35 | 0 | 0 |
| 242 | South America | -59.5432 | 13.1939 | 0 | 0 | 0 | 0 | 0 | 0 | 0 | 0 | 1 | 0 | 6 | 11 | 0 | 22 |
| 243 | South America | -58.75 | 5 | 11 | 13 | 18 | 31 | 0 | 43 | 0 | 28 | 65 | 23 | 19 | 4 | 15 | 16 |
| 244 | South America | -58.4438 | -23.4425 | 0 | 0 | 0 | 32 | 79 | 0 | 91 | 26 | 23 | 0 | 151 | 30 | 76 | 29 |
| 245 | South America | -56.0278 | 3.9193 | 6 | 8 | 17 | 0 | 7 | 6 | 16 | 29 | 33 | 19 | 50 | 5 | 21 | 33 |
| 246 | South America | -55.7658 | -32.5228 | 30 | 16 | 86 | 82 | 158 | 111 | 138 | 169 | 77 | 138 | 99 | 127 | 172 | 153 |
| 247 | South America | -51.9253 | -14.235 | 638 | 447 | 354 | 358 | 142 | 311 | 332 | 252 | 279 | 340 | 200 | 236 | 187 | 334 |

# **Appendix 2:** Investigation the effect of seasonal changes in the Northern Hemisphere on model performance during the third revision.

The proposed model was implemented to investigate the effect of seasonal changes in the Northern Hemisphere on model performance in two cases. In both ones, we used the COVID-19 data of the dataset, which cover Asia, Europe, North America, and parts of Africa in the Northern Hemisphere.

Case 1: The proposed model was trained using data from January 22, 2020 to August 31, 2020 and was evaluated on the data of the first two weeks of September (1 to 13).

In the second case, the proposed model was trained with data from January 22 to September 30, 2020 and evaluated on the data of the first two weeks of October (1 to 13).

The results of model implementation in these two cases are shown in Tables 1 and 2, respectively.

Table 1. Results of predicting the number of confirmed cases in the first two weeks of September 2020 on the dataset

| *Date* | *Continents* | | | | *Total number of predicted confirmed cases* | *Total number of Actual confirmed cases* | Percent Error |
| --- | --- | --- | --- | --- | --- | --- | --- |
|  | Africa | Asia | Europe | North America |  |  |  |
| 9/1/2020 | 6569 | 103804 | 19958 | 54832 | 185163 | 189796 | 2.44 |
| 9/2/2020 | 5344 | 116465 | 28080 | 67762 | 217651 | 200513 | 8.55 |
| 9/3/2020 | 4907 | 117439 | 29462 | 57733 | 209541 | 207064 | 1.20 |
| 9/4/2020 | 6552 | 116118 | 38137 | 63799 | 224606 | 221309 | 1.49 |
| 9/5/2020 | 6312 | 110792 | 29081 | 56839 | 203024 | 203490 | 0.23 |
| 9/6/2020 | 5461 | 122747 | 24160 | 44467 | 196835 | 187452 | 5.01 |
| 9/7/2020 | 3933 | 119112 | 44032 | 49978 | 217055 | 185758 | 16.85 |
| 9/8/2020 | 5860 | 119832 | 22355 | 61963 | 210010 | 198474 | 5.81 |
| 9/9/2020 | 6459 | 114628 | 24285 | 55319 | 200691 | 213799 | 6.13 |
| 9/10/2020 | 5768 | 112574 | 46692 | 67008 | 232042 | 223211 | 3.96 |
| 9/11/2020 | 5534 | 119069 | 31068 | 66545 | 222216 | 240548 | 7.62 |
| 9/12/2020 | 6649 | 116421 | 34060 | 60438 | 217568 | 217796 | 0.10 |
| 9/13/2020 | 5043 | 112974 | 28224 | 46340 | 192581 | 196282 | 1.89 |
| *Total* | 74391 | 1501975 | 399594 | 753023 | 2728983 | 2685492 | 1.62 |

Table 2. Results of predicting the number of confirmed cases in the first two weeks of October 2020 on the dataset

| *Date* | *Continents* | | | | *Total number of predicted confirmed cases* | *Total number of Actual confirmed cases* | Percent Error |
| --- | --- | --- | --- | --- | --- | --- | --- |
|  | Africa | Asia | Europe | North America |  |  |  |
| 10/1/2020 | 6517 | 129548 | 83054 | 51285 | 270404 | 249929 | 8.19 |
| 10/2/2020 | 7004 | 124389 | 109231 | 60049 | 300673 | 264623 | 13.62 |
| 10/3/2020 | 7771 | 120399 | 70212 | 59117 | 257499 | 242486 | 6.19 |
| 10/4/2020 | 7404 | 116983 | 62494 | 42635 | 229516 | 231703 | 0.94 |
| 10/5/2020 | 6268 | 111330 | 87066 | 94415 | 299079 | 257334 | 16.22 |
| 10/6/2020 | 6296 | 129020 | 68750 | 62517 | 266583 | 253093 | 5.33 |
| 10/7/2020 | 7753 | 120952 | 87128 | 51120 | 266953 | 283593 | 5.87 |
| 10/8/2020 | 6842 | 122291 | 102962 | 55676 | 287771 | 297698 | 3.33 |
| 10/9/2020 | 7183 | 123778 | 101519 | 63654 | 296134 | 298973 | 0.95 |
| 10/10/2020 | 6881 | 116208 | 85645 | 61025 | 269759 | 292215 | 7.68 |
| 10/11/2020 | 9457 | 118524 | 73248 | 57725 | 258954 | 246444 | 5.08 |
| 10/12/2020 | 5482 | 110454 | 94448 | 52305 | 262689 | 257289 | 2.10 |
| 10/13/2020 | 7090 | 124309 | 73273 | 56739 | 261411 | 280693 | 6.87 |
| *Total* | 91948 | 1568185 | 1099030 | 768262 | 3527425 | 3456073 | 2.06 |

To test whether the model takes into account seasonal changes, we examined the performance of the proposed model in predicting the number of cases in Iran (which is our country) and Ukraine in the first two weeks of October. The results of predicting the proposed model and comparing it with the actual values recorded for these two countries in the dataset are shown in Tables 3 and 4.

Table 3. Performance evaluation of the model to predict the number of cases in the first two weeks of October in Iran.

| Country,  (Latitude, Longitude) | Date | Actual | Predicted | Absolute Error | Percent Error |
| --- | --- | --- | --- | --- | --- |
| Iran  (32.427908, 53.688046) | 10/1/2020 | 3825 | 4193 | 368 | 9.62 |
|  | 10/2/2020 | 3552 | 4193 | 641 | 18.05 |
|  | 10/3/2020 | 3523 | 4193 | 670 | 19.02 |
|  | 10/4/2020 | 3653 | 4193 | 540 | 14.78 |
|  | 10/5/2020 | 3902 | 4193 | 291 | 7.46 |
|  | 10/6/2020 | 4151 | 4534 | 383 | 9.23 |
|  | 10/7/2020 | 4019 | 2928 | 1091 | 27.15 |
|  | 10/8/2020 | 4392 | 5332 | 940 | 21.40 |
|  | 10/9/2020 | 4142 | 3225 | 917 | 22.14 |
|  | 10/10/2020 | 3875 | 3679 | 196 | 5.06 |
|  | 10/11/2020 | 3822 | 4321 | 499 | 13.06 |
|  | 10/12/2020 | 4206 | 4330 | 124 | 2.95 |
|  | 10/13/2020 | 4108 | 3883 | 225 | 5.48 |
|  | 10/1/2020 | 4830 | 3478 | 1352 | 27.99 |
|  | Total | 56000 | 56675 | 675 | 1.21 |

Table 4. Performance evaluation of the model to predict the number of cases in the first two weeks of October in Ukraine.

| Country,  (Latitude, Longitude) | Date | Actual | Predicted | Absolute Error | Percent Error |
| --- | --- | --- | --- | --- | --- |
| Ukraine  (48.3794, 31.1656) | 10/1/2020 | 4179 | 4675 | 496 | 11.87 |
|  | 10/2/2020 | 4751 | 4723 | 28 | 0.59 |
|  | 10/3/2020 | 4785 | 4599 | 186 | 3.89 |
|  | 10/4/2020 | 4263 | 5067 | 804 | 18.86 |
|  | 10/5/2020 | 3905 | 4263 | 358 | 9.17 |
|  | 10/6/2020 | 4482 | 4471 | 11 | 0.25 |
|  | 10/7/2020 | 4887 | 4018 | 869 | 17.78 |
|  | 10/8/2020 | 5545 | 6576 | 1031 | 18.59 |
|  | 10/9/2020 | 5961 | 5271 | 690 | 11.58 |
|  | 10/10/2020 | 5901 | 4927 | 974 | 16.51 |
|  | 10/11/2020 | 4960 | 4172 | 788 | 15.89 |
|  | 10/12/2020 | 4606 | 4391 | 215 | 4.67 |
|  | 10/13/2020 | 5311 | 4675 | 636 | 11.98 |
|  | 10/1/2020 | 5780 | 4770 | 1010 | 17.47 |
|  | Total | 69316 | 66598 | 2718 | 3.92 |

Although time series usually need to be long enough (normally a few years) to adequately account for seasonality, based on the results of the model implementations, we believe that this model, even with that short a time series, is able to manage seasonality and can predict the number of cases with acceptable accuracy.

# Appendix 3: The performance evaluation of the proposed method in 10% of randomly selected regions in the data set in the first two weeks of October during the fourth revision.

In the previous report, we selected a country based on the simple random selection method, and investigated the performance of the proposed method on it to explore how the model handles the seasonality changes; the actual and prediction of confirmed cases were reported for this country (Ukraine) and our country as well.

In the present report, we randomly selected a country to investigate the performance of the model on another country and to address the seasonality concern. For this country, the actual and predicted cases during the first two weeks of October 2020, as well as the model percent error, are shown in Table 5.

Table 5. Performance evaluation of the model to predict the number of cases in the first two weeks of October in Russia.

| Country,  (Latitude, Longitude) | Date | Actual | Predicted | Absolute Error | Percent Error |
| --- | --- | --- | --- | --- | --- |
| Russia  (61.52401,105.3188) | 10/1/2020 | 8835 | 10244 | 1409 | 15.95 |
|  | 10/2/2020 | 9294 | 10244 | 950 | 10.22 |
|  | 10/3/2020 | 9735 | 8868 | 867 | 8.91 |
|  | 10/4/2020 | 10376 | 8868 | 1508 | 14.53 |
|  | 10/5/2020 | 10757 | 9593 | 1164 | 10.82 |
|  | 10/6/2020 | 11481 | 9593 | 1888 | 16.44 |
|  | 10/7/2020 | 10981 | 9593 | 1388 | 12.64 |
|  | 10/8/2020 | 11345 | 10058 | 1287 | 11.34 |
|  | 10/9/2020 | 11969 | 10447 | 1522 | 12.72 |
|  | 10/10/2020 | 12673 | 12097 | 576 | 4.55 |
|  | 10/11/2020 | 13442 | 11867 | 1575 | 11.72 |
|  | 10/12/2020 | 13406 | 10842 | 2564 | 19.13 |
|  | 10/13/2020 | 13690 | 10842 | 2848 | 20.80 |
|  | 10/14/2020 | 14041 | 11149 | 2892 | 20.60 |
|  | Total | 162025 | 144305 | 17720 | 10.94 |

In addition, to evaluate the performance of the model in different regions with different numbers of COVID-19 cases, we selected 10% of all countries in the Northern Hemisphere based on Stratified Sampling method. The reason for choosing this method is that in the proposed method, we divided the data set areas into three groups: areas with less than 200 confirmed cases per day, between 200 to 1000, and more than 1000 cases. This dataset contains information on 217 different regions in the Northern Hemisphere. Of these, 132 regions belong to the first group (less than 200 cases), 40 regions are in the second group and 45 regions are in the third group. Therefore, we randomly selected 10% of each group and evaluated the percent error of the model based on actual and predicted cases over a 14-day period (first two weeks of October, 2020). The results are presented in Table 6 and Figures 1 to 3.

Table 6. The performance of the model in 10% of countries randomly selected from the Northern Hemisphere.

| Region | Continents | Lat | Long | Actual | Predicted | PercentError | Group |
| --- | --- | --- | --- | --- | --- | --- | --- |
| Maldives | Asia | 3.2028 | 73.2207 | 771 | 844 | 9.47 | <200 |
| Guyana | South America | 4.86042 | -58.9302 | 695 | 635 | 8.63 | <200 |
| Sierra Leone | Africa | 8.46055 | -11.7799 | 84 | 90 | 7.14 | <200 |
| Guinea-Bissau | Africa | 11.8037 | -15.1804 | 65 | 72 | 10.77 | <200 |
| Martinique | Europe | 14.6415 | -61.0242 | 714 | 779 | 9.10 | <200 |
| Saint Barthelemy | Europe | 17.9 | -62.8333 | 19 | 18 | 5.26 | <200 |
| Guangxi | Asia | 23.8298 | 108.788 | 1 | 1 | 0.00 | <200 |
| Jiangsu | Asia | 32.9711 | 119.455 | 1 | 1 | 0.00 | <200 |
| Korea, South | Asia | 35.9078 | 127.767 | 1099 | 1052 | 4.28 | <200 |
| Inner Mongolia | Asia | 44.0935 | 113.945 | 4 | 4 | 0.00 | <200 |
| British Columbia | North America | 53.7267 | -127.648 | 1754 | 1701 | 3.02 | <200 |
| Estonia | Europe | 58.5953 | 25.0136 | 576 | 643 | 11.63 | <200 |
| Iceland | Europe | 64.9631 | -19.0208 | 1029 | 1088 | 5.73 | <200 |
| Venezuela | South America | 6.4238 | -66.5897 | 9883 | 10002 | 1.20 | 200<x<1000 |
| Guatemala | North America | 15.7835 | -90.2308 | 7348 | 7388 | 0.54 | 200<x<1000 |
| Luxembourg | Europe | 49.8153 | 6.1296 | 1521 | 1522 | 0.07 | 200<x<1000 |
| Norway | Europe | 60.472 | 8.4689 | 1926 | 1853 | 3.79 | 200<x<1000 |
| Bangladesh | Asia | 23.685 | 90.3563 | 19480 | 20164 | 3.51 | >1000 |
| US | North America | 40 | -100 | 689257 | 682913 | 0.92 | >1000 |
| Ontario | North America | 51.2538 | -85.3232 | 10033 | 9543 | 4.88 | >1000 |
| Austria | Europe | 47.5162 | 14.5501 | 13859 | 14852 | 7.17 | >1000 |
| Quebec | North America | 52.9399 | -73.5491 | 14706 | 14522 | 1.25 | >1000 |

Figure 1. The performance of the model for selected regions of the first group in the two-week period.

Figure 2. The performance of the model for selected regions of the second group in the two-week period.

Figure 3. The performance of the model for selected regions of the third group in the two-week period.

# Appendix 4: The results of the proposed method on the updated data during the second revision.

To find the best parameters of the proposed model, which includes determining the neighborhood and the interval of days whose information is effective in predicting the situation of the next day at any point, the algorithm was run on the data set containing information from January 22, 2020 to February 15, 2021. The data of the last two weeks (February 2-15, 2021) were used as a validation set and the rest were used as training data. The number of neighborhoods in the range between [0, 40] was examined. The interval between last 14 to 40 days was examined. The proposed algorithm then implemented all possible combinations of neighborhood and interval for three groups of areas with less than 200, between 200 and 1000 and more than 1000 cases per day.

The best parameters obtained by implementing the proposed model are shown in Table 7. According to data from the past year, the data set is rich enough for areas with less than 200 cases per day and has achieved 93% accuracy without using neighborhood information. For these areas, data between the last 28 to 14 days have been used to predict the number of cases in the next two weeks. For areas with 200 to 1000 cases per day, the proposed model has achieved 86.38% accuracy using the data of the last 14 to 15 days of the area itself and its 7 neighbors. For areas with more than 1,000 cases per day, the proposed algorithm, using a neighbor and information from the last 14 to 24 days, was able to achieve 99.56% prediction accuracy.

Due to the normalization of the MAE between [0, 1], this measure indicates the standard deviation of the prediction accuracy. As a result, the accuracy of the model is obtained from the following equation:

$$Accuracy=100-2\times{MAE}_{Test} (1)$$

Table 7. The results of the best models evaluated on COVID-19 dataset (January 22, 2020 to February 15,2021).

| Maximum Number of confirmed cases in a day | | Number of Neighbors | Interval of days [min,max] | MSE | MAE | Accuracy |
| --- | --- | --- | --- | --- | --- | --- |
| <200 | Train | - | [14,28] | 0.000956169 | 0.012399538 | 97.52% |
|  | Test |  |  | 0.00610283 | 0.034847613 | 93.03% |
| [200,1000) | Train | 7 | [14,15] | 0.001342893 | 0.020220526 | 95.96% |
|  | Test |  |  | 0.012803348 | 0.068124688 | 86.38% |
| ≥1000 | Train | 1 | [14,24] | 1.49E-06 | 0.000531365 | 99.89% |
|  | Test |  |  | 4.35E-05 | 0.002184404 | 99.56% |

After finding the optimal parameters, the optimal model was re-run on the validation data set and the number of cases was predicted for all 269 geographical regions in the dataset. It should be noted that the cases of Cruise ships are also recorded in the data set, which we did not include in the analysis. Table 8 shows the prediction of COVID-19 by the proposed model between 2 and 15 February 2021 in each continent, by classifying points by continent location.

Table 8. Forecast the COVID-19 new cases for the next two weeks (validation period).

| *Date* | *Continents* | | | | | | *Total number of predicted confirmed cases* |
| --- | --- | --- | --- | --- | --- | --- | --- |
|  | Africa | Asia | Australian | Europe | North America | South America |  |
| 2/2/2021 | 22497 | 61464 | 79 | 195828 | 133807 | 176213 | 589888 |
| 2/3/2021 | 20043 | 72626 | 157 | 216020 | 148747 | 102138 | 559731 |
| 2/4/2021 | 21866 | 72253 | 100 | 116896 | 117383 | 110998 | 439496 |
| 2/5/2021 | 19003 | 61166 | 12 | 193932 | 177525 | 88997 | 540635 |
| 2/6/2021 | 22509 | 52966 | 8 | 142312 | 178887 | 78497 | 475179 |
| 2/7/2021 | 16350 | 50736 | 7 | 119491 | 123236 | 59114 | 368934 |
| 2/8/2021 | 19217 | 56041 | 54 | 126034 | 107054 | 74473 | 382873 |
| 2/9/2021 | 20981 | 80835 | 79 | 123958 | 139518 | 119836 | 485207 |
| 2/10/2021 | 20966 | 66273 | 76 | 164940 | 163810 | 116708 | 532773 |
| 2/11/2021 | 24611 | 73287 | 118 | 159044 | 128552 | 93112 | 478724 |
| 2/12/2021 | 24216 | 77541 | 9 | 117825 | 136003 | 92077 | 447671 |
| 2/13/2021 | 18174 | 54559 | 41 | 88056 | 111837 | 91635 | 364302 |
| 2/14/2021 | 19431 | 72345 | 13 | 116259 | 115110 | 64701 | 387859 |
| 2/15/2021 | 13395 | 47278 | 41 | 109028 | 123173 | 54854 | 347769 |
| *Total* | 283259 | 899370 | 794 | 1989623 | 1904642 | 1323353 | 6401041 |

To compare the performance of the model, the prediction results are compared with the actual results and are shown in Table 9. For this evaluation, the Percent error criterion (Equation 5 in the manuscript) has been used. The error of the proposed model was 11.46% for a total of 14 days. Also, the continental prevalence rate according to the Equation 6 in the manuscript is shown in Figure 4.

The results show that despite the fact that the proposed model is based only on the first 68 days of COVID-19 outbreak in the world, but now, almost a year after the introduction of the model, it still has high accuracy in predicting the incidence of COVID-19. It is worth noting that, in the past year, in addition to applying various restrictions and policies, vaccination against the virus has also been carried out. Also, the proposed model, using neighborhood information, indirectly uses the effective policies of other regions in predicting the incidence of COVID-19 in each region. This suggests that the proposed model is general and can be used as a tool for health policymakers to predict not only the incidence of COVID-19 but also future epidemics.

In such cases of serious public health problems, with many unknowns expected from a new epidemic, models that can predict prevalence can guide planning and resource allocation for prevention, treatment and palliative care.


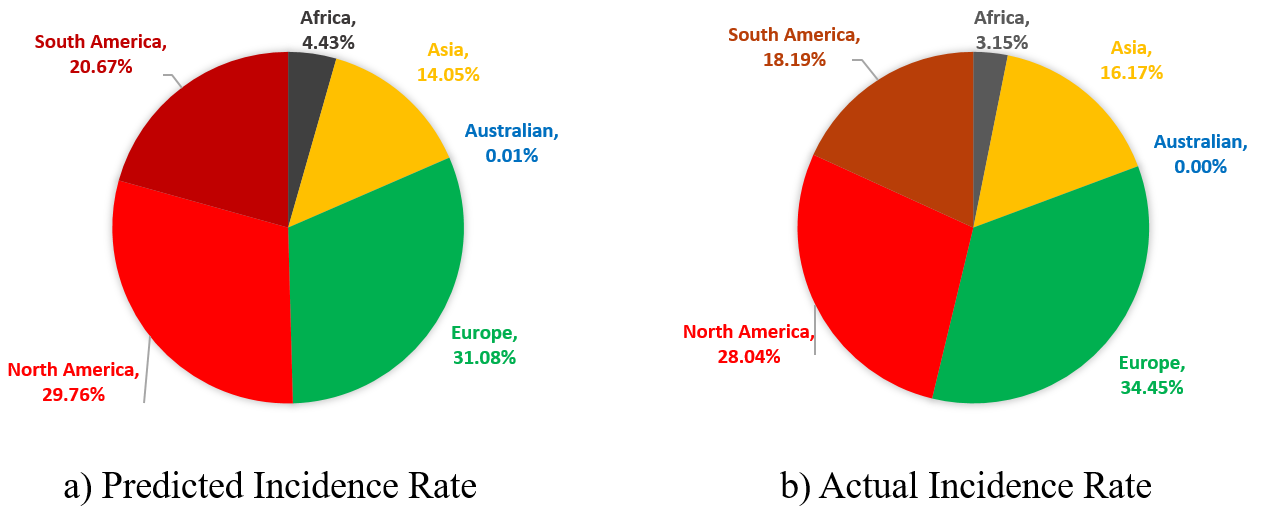


Figure 4. Comparison of predicted and actual continental incidence rates between February 2 and 15, 2021.

Table 9. Comparison of predicted and actual daily incidence of COVID-19.

| *Date* |  | | *Across all 269 geographic regions* | |  | *Percent error* |
| --- | --- | --- | --- | --- | --- | --- |
|  |  | | Predicted | Actual |  |  |
| 2/2/2021 |  | 589888 | | 456829 |  | 29.13% |
| 2/3/2021 |  | 559731 | | 522009 |  | 7.23% |
| 2/4/2021 |  | 439496 | | 466550 |  | 5.80% |
| 2/5/2021 |  | 540635 | | 534584 |  | 1.13% |
| 2/6/2021 |  | 475179 | | 372129 |  | 27.69% |
| 2/7/2021 |  | 368934 | | 397806 |  | 7.26% |
| 2/8/2021 |  | 382873 | | 315478 |  | 21.36% |
| 2/9/2021 |  | 485207 | | 427546 |  | 13.49% |
| 2/10/2021 |  | 532773 | | 435053 |  | 22.46% |
| 2/11/2021 |  | 478724 | | 440868 |  | 8.59% |
| 2/12/2021 |  | 447671 | | 409074 |  | 9.44% |
| 2/13/2021 |  | 364302 | | 348470 |  | 4.54% |
| 2/14/2021 |  | | 387859 | 277853 |  | 39.59% |
| 2/15/2021 |  | | 347769 | 338612 |  | 2.70% |
| *Total number of confirmed cases* |  | | 6401041 | 5742861 |  | **11.46%** |
